# Loss of E-Cadherin Leads to Druggable Vulnerabilities in Sphingolipid Metabolism and Vesicle Trafficking

**DOI:** 10.3390/cancers14010102

**Published:** 2021-12-26

**Authors:** Tom Brew, Nicola Bougen-Zhukov, Wilson Mitchell, Lyvianne Decourtye, Emily Schulpen, Yasmin Nouri, Tanis Godwin, Parry Guilford

**Affiliations:** Cancer Genetics Laboratory, Centre for Translational Cancer Research (Te Aho Matatū), Department of Biochemistry, University of Otago, Dunedin 9016, New Zealand; tom.p.brew@gmail.com (T.B.); nicola.bougen-zhukov@otago.ac.nz (N.B.-Z.); mitwi509@student.otago.ac.nz (W.M.); lyvianne.decourtye@otago.ac.nz (L.D.); schem499@student.otago.ac.nz (E.S.); ynouri@malaghan.org.nz (Y.N.); tanis.godwin@otago.ac.nz (T.G.)

**Keywords:** hereditary diffuse gastric cancer, E-cadherin, synthetic lethality, chemoprevention, endocytosis, autophagy, sphingolipid metabolism

## Abstract

**Simple Summary:**

Germline loss of the *CDH1* gene is the primary genetic basis for hereditary diffuse gastric cancer, a disease resulting in elevated risk of both diffuse gastric cancer and lobular breast cancer. Current preventative treatment consists of prophylactic total gastrectomy, a therapy with several associated long-term morbidities. To address the lack of targeted molecular therapies for hereditary diffuse gastric cancer, we have utilized a synthetic lethal approach to identify candidate compounds that can specifically kill *CDH1*-null cells. Inhibitors of sphingolipid metabolism and vesicle trafficking pathways were identified as promising candidate compounds in a cell line model of *CDH1* loss, then further validated in murine-derived organoid models of hereditary diffuse gastric cancer. With further research, these findings may lead to the development of novel chemoprevention strategies for the treatment of hereditary diffuse gastric cancer.

**Abstract:**

Germline inactivating variants of *CDH1* are causative of hereditary diffuse gastric cancer (HDGC), a cancer syndrome characterized by an increased risk of both diffuse gastric cancer and lobular breast cancer. Because loss of function mutations are difficult to target therapeutically, we have taken a synthetic lethal approach to identify targetable vulnerabilities in *CDH1*-null cells. We have previously observed that *CDH1*-null MCF10A cells exhibit a reduced rate of endocytosis relative to wildtype MCF10A cells. To determine whether this deficiency is associated with wider vulnerabilities in vesicle trafficking, we screened isogenic MCF10A cell lines with known inhibitors of autophagy, endocytosis, and sphingolipid metabolism. Relative to wildtype MCF10A cells, *CDH1^−/−^* MCF10A cells showed significantly greater sensitivity to several drugs targeting these processes, including the autophagy inhibitor chloroquine, the endocytosis inhibitors chlorpromazine and PP1, and the sphingosine kinase 1 inhibitor PF-543. Synthetic lethality was confirmed in both gastric and mammary organoid models of *CDH1* loss, derived from *CD44*-Cre/*Cdh1*^fl/fl^/tdTomato mice. Collectively, these results suggest that both sphingolipid metabolism and vesicle trafficking represent previously unrecognised druggable vulnerabilities in *CDH1*-null cells and may lead to the development of new therapies for HDGC.

## 1. Introduction

Hereditary diffuse gastric cancer (HDGC) is an autosomal dominant syndrome, primarily characterized by extremely elevated risks of both diffuse-type gastric cancer (DGC) and lobular breast cancer. Men and women have an approximate lifetime gastric cancer risk of 42–70% and 33–56%, respectively, and women have a lifetime lobular breast cancer risk of 39–55% [1]. Pathogenic germline variants in *CDH1* are the primary genetic cause of HDGC [2]. However, pathogenic *CTNNA1* variants have been recently accepted as causative of HDGC in a minority of families [1]. Early-stage DGC is usually asymptomatic, and although regular endoscopic screening reduces the risk of progressive disease, surveillance is not without risk [1]. Following the invasion of the *muscularis propria*, disease progression is rapid, greatly limiting treatment options [3]. The only available preventative treatment for HDGC is prophylactic total gastrectomy, which is recommended for individuals harboring pathogenic *CDH1* mutations that are at least 20 years old [1]. The risk of gastric cancer is eliminated if surgery is performed at an early age. However, there are several long-term co-morbidities associated with treatment, most commonly consisting of diarrhea, weight loss and dumping syndrome [1]. The high penetrance of HDGC, combined with the high morbidity of current preventative treatments, necessitates the development of chemoprevention approaches that can exploit vulnerabilities in *CDH1*-null cells.

*CDH1* is a tumor suppressor gene encoding the transmembrane protein E-cadherin, which is primarily localized to the epithelial basolateral membrane, and constitutes a critical component of adherens junctions [4]. E-cadherin undergoes homophilic ligation with adjacent cells to maintain cell–cell adhesion, plays a role in essential cell signaling pathways and interacts with the actin cytoskeleton through the cadherin–catenin complex [5,6]. In addition to their role in HDGC, *CDH1* mutations are commonly reported in sporadic DGC and lobular breast cancer [7,8], and miRNA-mediated suppression of *CDH1* has been reported in intestinal-type gastric cancer [9,10].

Because tumor suppressor proteins, such as E-cadherin, are inactivated or lost in cancer, they cannot be targeted directly for therapeutic benefit. However, the concept of synthetic lethality can address this difficulty. Synthetic lethality describes a relationship between two genes whereby the loss of function in either gene maintains cell viability, but the simultaneous loss of both genes induces cell death [11]. In the context of HDGC, synthetic lethal partner genes of *CDH1* can become actionable drug targets, thus resulting in preferential death of the *CDH1*-null tumor cells. Our laboratory has previously performed a genome-wide siRNA screen, a large unknown compound screen, and targeted drug screening to identify synthetic lethal partner genes for *CDH1* in an isogenic pair of MCF10A cell lines, one presenting with abrogated *CDH1* expression [12,13,14,15,16]. MCF10A cells are a non-tumorigenic breast-derived cell line exhibiting few background mutations and a relatively stable genome [17,18]. In the absence of a suitable non-malignant gastric cell line, MCF10A was selected for these studies because of the importance of a relatively normal background genotype for chemoprevention studies. Additionally, the use of a breast-derived cell line may aid in the development of novel treatment strategies for the *CDH1^−/−^* lobular breast cancer component of HDGC. To complement this isogenic breast cell line, our laboratory has recently established a *CDH1* isogenic NCI-N87 gastric cancer cell line pair [13] and murine-derived gastric and mammary organoid models of inducible *Cdh1* loss.

We have previously shown that the loss of *CDH1* results in a disorganized cell cytoskeleton [14] and that *CDH1^−/−^* cells exhibit a decreased rate of endocytosis [15]. Combined with genome-wide siRNA screening in *CDH1*^+/+^ and *CDH1*^−/−^ cells [16], and analysis of expression patterns correlated with *CDH1* levels in TCGA gastric cancer datasets [15], we predicted that this might reflect wider perturbations to membrane organization and vesicle trafficking. To test this hypothesis, we assessed inhibitors of endocytosis, autophagy, intracellular trafficking, and plasma membrane organization in an MCF10A model of HDGC. Promising compounds capable of preferentially inhibiting *CDH1^−/−^* cell growth were subsequently assessed in isogenic NCI-N87 cell lines and in murine-derived gastric and breast organoids with inducible *Cdh1* loss. These findings represent previously unidentified druggable vulnerabilities in *CDH1^−/−^* cells and may lead to the development of a novel chemopreventative approach for the management of HDGC, or novel therapies for sporadic E-cadherin-deficient cancers.

## 2. Materials and Methods

### 2.1. Cell Culture

MCF10A and MCF10A *CDH1^−/−^* isogenic cell lines were purchased from Sigma-Aldrich (#CLLS1042, Sigma-Aldrich, St Louis, MO, USA). Cells were cultured in Dulbecco’s Modified Eagle Medium/Nutrient Mixture F-12 with GlutaMAX (Thermo Fisher Scientific, Waltham, MA, USA) supplemented with 5% horse serum (Invitrogen, Carlsbad, CA, USA), 20 ng/mL EGF (Peprotech, Rehovot, Israel), 100 ng/mL cholera toxin (Sigma-Aldrich, St Louis, MO, USA), 0.5 µg/mL hydrocortisone (Sigma-Aldrich, St Louis, MO, USA) and 10 µg/mL insulin (Novo Nordisk Pharmaceuticals Ltd., Bagsværd, Denmark).

The NCI-N87 cell line was purchased from ATCC, and the NCI-N87 *CDH1^−/−^* cell line was generated within our laboratory [13]. Cells were grown in Dulbecco’s Modified Eagle Medium/Nutrient Mixture F-12 with GlutaMAX (Thermo Fisher Scientific, Waltham, MA, USA) supplemented with 10% fetal bovine serum (Invitrogen, Carlsbad, CA, USA).

All cells were cultured in a 37 °C humidified incubator at 5% CO_2_.

### 2.2. Drug Screening

For the 3-point drug screen, MCF10A and MCF10A *CDH1^−/−^* cells were seeded at 1000 cells per well in 384-well, black-walled, clear-bottom tissue culture plates (Greiner Bio-One, Frickenhausen, Germany) in 30 µL of complete growth medium. After 24 h of growth, to confirm sufficient seeding accuracy, outer wells were stained with 1 µg/mL Hoechst 33342 in PBS, incubated for 2 h at room temperature in the dark, then nuclei were counted using a Cytation 5 Cell Imaging Multi-Mode Reader (Biotek, Winooski, VT, USA). If the ratio of MCF10A:MCF10A *CDH1^−/−^* cells was between 0.65 and 1.3, plates were treated with 10 µL of compound or respective vehicle control as previously described [19], then incubated for a further 48 h. Cells were fixed and stained with 0.25% paraformaldehyde, 0.075% saponin and 1 µg/mL of Hoechst 33342 in PBS, incubated overnight at room temperature in the dark, then nuclei were enumerated using the Cytation 5 Cell Imaging Multi-Mode Reader (Biotek, Winooski, VT, USA). All automated imaging captured four fields per well at 4× magnification, with nuclei counts normalized to the respective vehicle control for each cell line. IC_50_ values were calculated using CompuSyn software. All experiments were performed in biological duplicates.

For subsequent 9-point drug screening, identical methodology was utilized, with the following modifications. MCF10A and NCI-N87 isogenic cell lines were seeded at 4000 and 10,000 cells per well, respectively. MCF10A cells were seeded in 96-well, black-walled, clear-bottom tissue culture plates (Corning, Corning, NY, USA) in 90 µL of complete growth medium. NCI-N87 cells were seeded at 2500 cells per well in 384-well, black-walled, clear-bottom tissue culture plates (Greiner Bio-One, Frickenhausen, Germany). Automated imaging captured six fields per well at 4× magnification on the Cytation 5 Cell Imaging Multi-Mode Reader (Biotek, Winooski, VT, USA). A pilot screen of nine drug concentrations was assessed, with the dilution series adjusted to investigate a range of concentrations capable of inhibiting cell growth. With the exception of the pilot screen, all experiments were performed in biological triplicates.

For assessing compound synergy in MCF10A and MCF10A *CDH1^−/−^* cells, identical methodology to the 9-point drug screening was utilized, with the following modifications. After 24 h of growth, cells were treated with 10 µL of either drug 1 and vehicle control for drug 2, drug 2 and vehicle control for drug 1, or drugs 1 and 2 in combination. Combination index values were calculated using CompuSyn software. All combination treatments were performed as biological triplicates. Single-agent treatment data was generated from a minimum of 16 biological replicates, with at least two measurements originating from each plate that included the compound as part of a combination treatment. This pooled data enabled standardised 9-point MCF10A WT and *CDH1^−/−^* viability measurements, thus enabling accurate determination of combination synergism.

A full list of compounds assessed in this study can be found in Table S1.

### 2.3. Autophagy Assay

Cells were seeded into 6-well tissue culture plates (Corning, Corning, NY, USA) in 2 mL of complete growth medium at densities of 0.5 × 10^5^ and 0.65 × 10^5^ for MCF10A and MCF10A *CDH1^−/−^* cells, or 1.5 × 10^5^ and 2 × 10^5^ cells for NCI-N87 and NCI-N87 *CDH1^−/−^* cells, respectively. After 24 h of growth, cells were treated with 0.2% DMSO. After a further 48 h for MCF10A, or 72 h for NCI-N87, cells were harvested, and the autophagy assay was performed according to the manufacturer’s instructions (Autophagy Assay Kit, Abcam, Cambridge, UK). Flow cytometry was performed on a BD Fortessa Flow Cytometer (BD Biosciences, San Jose, CA, USA).

### 2.4. Organoid Culture

Gastric organoids were cultured as described elsewhere, with minor modifications [20]. Briefly, antral glands were extracted from the stomachs of 6-8 week old mice and embedded in 50 µL of Matrigel (Corning, Corning, NY, USA) per well in a 24-well culture plate (Greiner Bio-One, Frickenhausen, Germany). Cultures were submerged in 500 µL of complete growth medium consisting of Advanced Dulbecco’s Modified Eagle Medium/Ham’s F-12 Nutrient Mix (Thermo Fisher Scientific, Waltham, MA, USA) supplemented with 10 mM HEPES (Thermo Fisher Scientific, Waltham, MA, USA), 2 mM GlutaMAX (Thermo Fisher Scientific, Waltham, MA, USA), 100 µg/mL Primocin (Invivogen, San Diego, CA, USA), 1 mM N-acetyl-L-cysteine (Sigma-Aldrich, St Louis, MO, USA), 10 nM [Leu^15^]-Gastrin I (Sigma-Aldrich, St Louis, MO, USA), 50 ng/mL EGF (Peprotech, Rehovot, Israel), 100 ng/mL FGF10 (Thermo Fisher Scientific, Waltham, MA, USA), 10 µM Y-27632 (Sigma-Aldrich, St Louis, MO, USA), 2 µM A 83-01 (Sapphire North America, Ann Arbor, MI, USA), 1× B-27 supplement (Thermo Fisher Scientific, Waltham, MA, USA), 1× N-2 supplement (Thermo Fisher Scientific, Waltham, MA, USA), 10% R-spondin 1-conditioned medium, 10% noggin-conditioned medium and 50% Wnt3a-conditioned medium. Primocin was only utilized following gland isolation from mice, and was replaced in subsequent growth medium with 1× penicillin-streptomycin (Thermo Fisher Scientific, Waltham, MA, USA).

Culture of mammary organoids was adapted from a method described by Ewlad et al. [21]. Mammary glands were extracted from virgin female mice and embedded in 80 µL of Matrigel (Corning, Corning, NY, USA) per well in a 24-well culture plate (Greiner Bio-One, Frickenhausen, Germany). A total of 500 µL of complete growth medium was then added to cultures, consisting of Advanced Dulbecco’s Modified Eagle Medium/Ham’s F-12 Nutrient Mix (Thermo Fisher Scientific, Waltham, MA, USA) supplemented with 10 mM HEPES (Thermo Fisher Scientific, Waltham, MA, USA), 2 mM GlutaMAX (Thermo Fisher Scientific, Waltham, MA, USA), 1× penicillin-streptomycin (Thermo Fisher Scientific, Waltham, MA, USA), 1.25 mM N-acetyl-L-cysteine (Sigma-Aldrich, St Louis, MO, USA), 50 ng/mL EGF (Peprotech, Rehovot, Israel), 10 ng/mL FGF10 (Thermo Fisher Scientific, Waltham, MA, USA), 5 µM Y-27632 (Sigma-Aldrich, St Louis, MO, USA), 1 µM A 83-01 (Sapphire North America, Ann Arbor, MI, USA), 1× B-27 supplement (Thermo Fisher Scientific, Waltham, MA, USA), 5 µg/mL insulin (Sigma-Aldrich, St Louis, MO, USA), 100 ng/mL hydrocortisone (Sigma-Aldrich, St Louis, MO, USA), 5 ng/mL FGF2 (Peprotech, Rehovot, Israel), 0.5% R-spondin 1-conditioned medium, and 2.5% Wnt3a-conditioned medium.

Conditioned growth medium was generated from L Wnt-3a, HA-R-Spondin1-Fc 293T and HEK-293 Noggin-Fc cells as described elsewhere [22]. L Wnt-3a and HEK-293 Noggin-Fc cells were generated by the Clevers laboratory (Utrecht, Netherlands), and were provided by the Vincan laboratory (Melbourne, Australia). HA-R-Spondin1-Fc 293T cells were purchased from Trevigen (Trevigen, Gaithersburg, MD, USA).

Gastric organoids were passaged every 6–7 days. Physical disruption of organoid structure was achieved via aspiration through a 20G needle, followed by incubation in 0.05% trypsin-EDTA solution (Thermo Fisher Scientific, Waltham, MA, USA) for 10 min at 37 °C to generate a single-cell suspension. A total of 1000 gastric organoid cells were seeded per well and cultured as described above for antral glands. Mammary organoids were passaged every 7–9 days through incubation in 0.25% trypsin-EDTA solution (Thermo Fisher Scientific, Waltham, MA, USA) for 15 min at 37 °C to isolate single cells. A total of 3000 cells were seeded per well, and cultured as described above for mammary glands.

Organoids were generated from two transgenic mouse models, both originating from a C57 Black 6 background: *CD44*-Cre/*Cdh1*^fl/fl^/tdTomato mice and *CD44*-Cre/tdTomato mice (engineered by Ozgene, Perth, Australia). All animal procedures were approved by the University of Otago Animal Welfare and Ethics Committee (DET35/15 and AUP-19-149) and were performed in accordance with University of Otago guidelines and regulations.

### 2.5. Fluorescence-Activated Cell Sorting

*CD44*-Cre/*Cdh1*^fl/fl^/tdTomato and *CD44*-Cre/tdTomato organoids were cultured for 24 h, then Cre recombinase activity was induced with 1 µM endoxifen (Sigma-Aldrich, St Louis, MO, USA). After an additional four days of culture, organoids were passaged and resuspended in 1 mL of filter-sterilised fluorescence-activated cell sorting buffer comprising 2 mM EDTA and 1% fetal bovine serum (Scharlau, Barcelona, Spain) in PBS, pH-adjusted to 7.2. A total of 12.5 µL of Matrigel (Corning, Corning, NY, USA) was dispensed into each well of a 96-well, black-walled, clear-bottom tissue culture plate (Corning, Corning, NY, USA) on ice. Fluorescence-activated cell sorting was performed on a BD FACSAria™ Fusion Cell Sorter (BD Biosciences, San Jose, CA, USA) to sort and dispense 20 individual tdTomato-positive cells into each well of the 96-well plate. Organoids were cultured from single cells for a period of 11 days and monitored via brightfield microscopy on a Nikon Eclipse Ti inverted microscope (Nikon, New York City, NY, USA), with images captured by a DS-QiMc camera (Nikon, New York City, NY, USA).

### 2.6. Immunofluorescence

*CD44*-Cre/*Cdh1*^fl/fl^/tdTomato and *CD44*-Cre/tdTomato organoid cells were seeded on cover slips (Menzel-Glaser, Bad Wildungen, Germany) in a 24-well culture plate (Greiner Bio-One, Frickenhausen, Germany) in 12.5 µL of Matrigel (Corning, Corning, NY, USA). Organoids were cultured for 24 h, then Cre recombinase activity was induced with 1 µM endoxifen (Sigma-Aldrich, St Louis, MO, USA). After four days of growth, organoids were washed with PBS three times, then fixed with 4% paraformaldehyde for 30 min at room temperature. Organoids were washed with PBS three times, then incubated in 500 µL of blocking buffer, consisting of 10% horse serum (Invitrogen, Carlsbad, CA, USA) and 0.5% Triton™ X-100 (Sigma-Aldrich, St Louis, MO, USA) in PBS for 1 h at room temperature, in the dark. Organoids were washed with PBS, then incubated in PBS supplemented with 10% horse serum (Invitrogen, Carlsbad, CA, USA), 2% fetal bovine serum (Invitrogen, Carlsbad, CA, USA), 0.1% Triton™ X-100 (Sigma-Aldrich, St Louis, MO, USA) and 1:100 anti-E-cadherin antibody (goat origin, #AF748, R&D Systems, Minneapolis, MN, USA) for 2 h at room temperature, in the dark. Organoids were washed with PBS three times, then incubated in PBS supplemented with 10% horse serum (Invitrogen, Carlsbad, CA, USA), 2% fetal bovine serum (Invitrogen, Carlsbad, CA, USA), and 1:1000 anti-goat Alexa Fluor 488 antibody (donkey origin, #A11055, Invitrogen, Carlsbad, CA, USA) for 2 h at room temperature, in the dark. Organoids were washed with PBS five times, then bridge-mounted on Fisherbrand™ Superfrost™ Plus microscope slides (Thermo Fisher Scientific, Waltham, MA, USA). Two 22 × 22 mm coverslips (Menzel-Glaser, Bad Wildungen, Germany) were attached to either end of the slide using transparent nail varnish, then two drops of ProLong™ Gold Antifade Mountant with DAPI (Invitrogen, Carlsbad, CA, USA) was added between the two cover slips. Cover slips containing organoids were transferred to the microscope slide and placed Matrigel side-down. Samples were incubated for 30 min at room temperature to enable ProLong™ Gold Antifade Mountant with DAPI (Invitrogen, Carlsbad, CA, USA) to dry. Transparent nail varnish was applied around the edges of all three cover slips to ensure an air-tight seal. The resulting microscope slide consisted of two supporting coverslips at either end to elevate the coverslip containing organoids, alleviating downward pressure on the organoid sample and enabling maintenance of 3-dimensional structure. Confocal microscopy was performed on an Olympus Fluoview FV1000 Confocal Microscope (Olympus, Auckland, New Zealand), with 43–98 images captured per organoid, dependent on size.

### 2.7. Organoid Drug Screening

Gastric organoids were seeded at 1000 cells per well in 96-well, black-walled, clear-bottom tissue culture plates (Corning, Corning, NY, USA) in 50 µL of Matrigel (Corning, Corning, NY, USA) and 100 µL of complete growth medium. Mammary organoids were seeded at 300 cells per well in 384-well, black-walled, clear-bottom tissue culture plates (Corning, Corning, NY, USA) in 12.5 µL of Matrigel (Corning, Corning, NY, USA) and 20 µL of complete growth medium. Gastric organoids were cultured for 24 h, then Cre recombinase activity was induced with 1 µM endoxifen (Sigma-Aldrich, St Louis, MO, USA). Mammary organoids were cultured for 72 h, then treated with 0.5 µM endoxifen (Sigma-Aldrich, St Louis, MO, USA). After a further 24 h of culture, gastric organoids were treated with 100 µL of compound across a 3-point dilution series or respective vehicle controls. Mammary organoids were instead treated after an additional 48 h of culture with 40 µL of compound or respective vehicle control. After 48 h of drug treatment, brightfield microscopy was performed on a Nikon Eclipse Ti inverted microscope (Nikon, New York City, NY, USA), with images of gastric organoids captured across the entire surface area of each well, and across multiple focal planes, by a DS-QiMc camera (Nikon, New York City, NY, USA). A single focal plane that passed through the approximate centre of each organoid was utilized for measuring organoid area in ImageJ software [23]. Typical images obtained using this approach are depicted in Figure S1. An average of 32 gastric organoids were imaged per well. Mammary organoids were instead imaged on the Cytation 5 imaging reader (Biotek, Winooski, VT, USA), using the RFP channels. Four regions were captured per well, with 11 Z-stacks per image. Images were stitched together, and Z-stacks were combined to generate a single merged image per well. Mammary organoid area was calculated from RFP images using the Cytation 5 software (Biotek, Winooski, VT, USA). For both gastric and mammary cultures, average organoid size was calculated from triplicate wells for each condition and normalized to the respective vehicle control for each organoid line. All experiments were performed in biological triplicates.

## 3. Results

### 3.1. Identification of Novel Synthetic Lethal Pathways for CDH1

**Figure 1:**
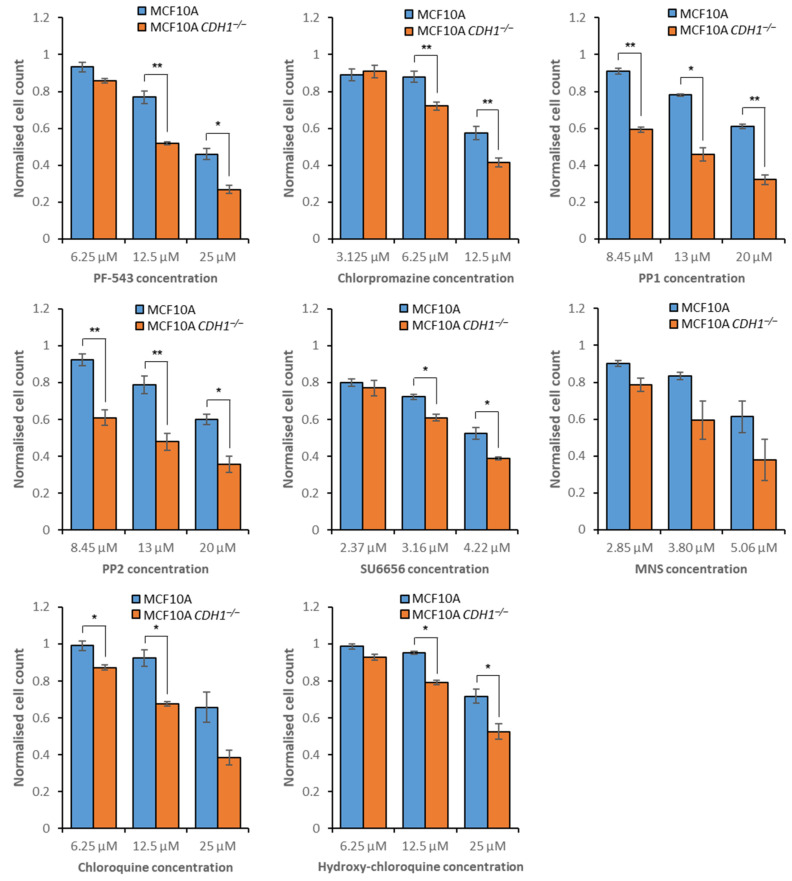
Compounds classified as synthetic lethal from the initial drug screen. MCF10A and MCF10A *CDH1^−/−^* cells were drugged, then viability was quantified through nuclei counting, and normalization to vehicle controls. Although a nine-point serial dilution was assessed, only the three consecutive compound concentrations that exhibited the greatest difference in viability between *CDH1*^+/+^ and *CDH1^−/−^* cells are depicted. MCF10A *CDH1^−/−^* cells were more sensitive to inhibitors of sphingolipid metabolism (PF-543), clathrin-mediated endocytosis (chlorpromazine), flotillin-mediated endocytosis (PP1, PP2 and SU6656), vesicle formation (MNS) and autophagy (chloroquine and hydroxy-chloroquine). Average values were calculated from three biological replicates, with +/− 1 standard error of the mean depicted by error bars. *P*-values were calculated using Student’s *t*-test; * *p* ≤ 0.05, ** *p* ≤ 0.01. MNS: 3,4-Methylenedioxy-β-nitrostyrene.

To assess potential vulnerabilities in *CDH1^−/−^* cells, we screened MCF10A and MCF10A *CDH1^−/−^* cells with 26 inhibitors targeting endocytosis, autophagy, intracellular vesicle trafficking and plasma membrane organization (Table 1). Initial drug screening consisted of a 3-point serial dilution of each compound in a higher-throughput screening format (data not shown). Subsequent validation was performed across a 9-point serial dilution to both validate the initial screening results and optimize dosage. Utilizing equivalent thresholding to our laboratory’s previous genome-wide siRNA screen [16], compounds were classified as synthetic lethal if any assessed concentration resulted in a ≥15% reduction in MCF10A *CDH1^−/−^* cell numbers relative to MCF10A cells, with MCF10A cells maintaining at least 50% viability. A total of 8 of the 26 assessed compounds were classified as synthetic lethal, with targets enriched across sphingolipid metabolism, clathrin- and flotillin-mediated endocytosis, and autophagy (Figure 1, Tables S2 and S3). To further refine the underlying mechanisms of synthetic lethality and potentially lead to more specific inhibition of *CDH1^−/−^* cells, additional drug screening was performed to explore each pathway of interest in greater detail, as described below.

### 3.2. MCF10A CDH1^−/−^ Cells Are Vulnerable to the Inhibition of Sphingolipid Metabolism and Signaling

MCF10A *CDH1^−/−^* cells were more sensitive to treatment with PF-543, a potent sphingosine kinase 1 inhibitor, than MCF10A cells. In contrast, neither cell line was significantly more sensitive to ABC294640, a sphingosine kinase 2 inhibitor. Sphingosine kinases are enzymes responsible for phosphorylating sphingosine into sphingosine-1-phosphate [46]. Because potential redundancy between the function of sphingosine kinase 1 and sphingosine kinase 2 has been proposed in the literature [47], we assessed three pan-sphingosine kinase inhibitors (SKI 178, MP A08 and SLC5111312) [48] to determine if greater synthetic lethality would be observed. However, SKI 178 and MP A08 induced indiscriminate growth inhibition, and SLC5111312 was only more potent against MCF10A *CDH1^−/−^* cells at a single concentration (Tables S4 and S5). In NCI-N87 cells, a gastric cancer cell line with a highly dysregulated genome, synthetic lethality between *SPHK1* and *CDH1* was abrogated, demonstrating the importance of genetic background to *SPHK1*′s synthetic lethality (Figure S2A, Tables S12 and S13).

**Figure 2:**
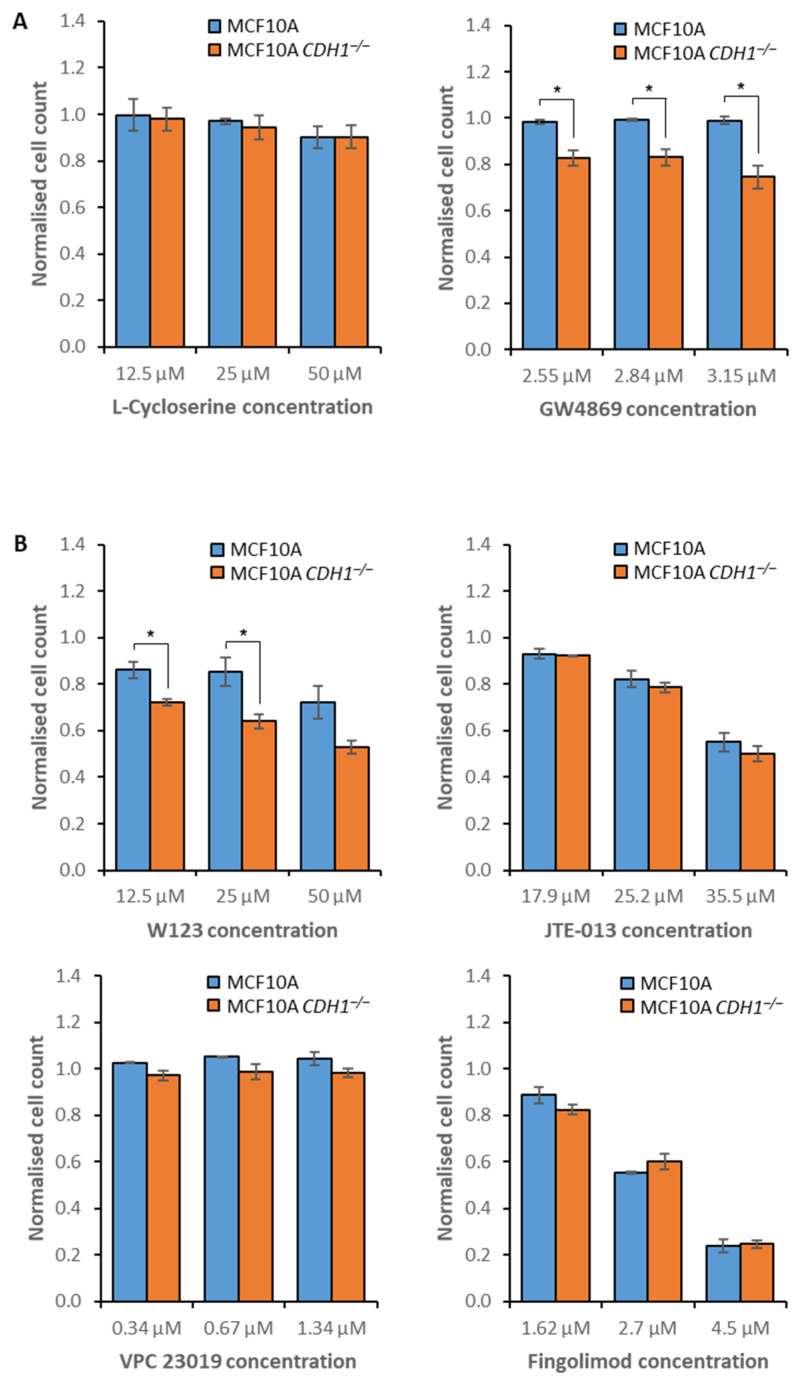
Efficacy of compounds inhibiting sphingolipid metabolism and signaling. MCF10A and MCF10A *CDH1^−/−^* cells were drugged, then viability was quantified through nuclei counting, and normalization to vehicle controls. Although a nine-point serial dilution was assessed, only the three consecutive compound concentrations that exhibited the greatest difference in viability between *CDH1*^+/+^ and *CDH1^−/−^* cells are depicted. Where no toxicity was observed, the maximum assessed concentrations are depicted. (**A**) Cell viability following treatment with inhibitors of sphingolipid metabolism. (**B**) Cell viability following treatment with inhibitors of S1PRs. Average values were calculated from three biological replicates, with +/− 1 standard error of the mean depicted by error bars. *P*-values were calculated using Student’s *t*-test; * *p* ≤ 0.05.

To further explore this potential vulnerability, two additional inhibitors of sphingolipid metabolism were assessed in MCF10A cells: L-cycloserine and GW4869. L-cycloserine inhibits 3-ketodhihydrosphingosine synthetase, the enzyme responsible for initiating sphingolipid synthesis [49]. Only minimal toxicity was observed following treatment, with no differential between the isogenic cell lines (Figure 2A, Tables S4 and S5). GW4869 inhibits neutral sphingomyelinase, which converts sphingomyelin into ceramide, the precursor for sphingosine [50]. GW4869 treatment inhibited the growth of MCF10A *CDH1^−/−^* cells across a range of concentrations but caused no growth inhibition of *CDH1*^+/+^ cells (Figure 2A, Tables S4 and S5).

Sphingosine-1-phosphate can be secreted from the cell for subsequent binding to any of the five G protein-coupled receptors, sphingosine-1-phosphate receptors 1–5 (S1PR1–5), on the surface of either the secreting cell or surrounding cells, with downstream function dependent on the bound receptor [51,52]. To determine if the underlying synthetic lethality with sphingosine kinase 1 was due to decreased S1PR agonism, we assessed several inhibitors of these receptors in MCF10A cells. W123 inhibits S1PR1, JTE-013 inhibits S1PR2, VPC 23019 inhibits S1PR1 and S1PR3, and fingolimod is a pan-S1PR inhibitor [53,54,55,56]. Although S1PR1-3 are expressed ubiquitously, S1PR4 and S1PR5 are only expressed in lymphoid tissue and the central nervous system respectively [57,58], and thus were not investigated in the context of HDGC. *CDH1^−/−^* cells showed increased sensitivity to W123 treatment across several concentrations, and VPC 23019 did not induce any reduction in cell numbers. However, it should be noted that VPC 23019 has particularly limited solubility and could only be tested at a maximum concentration of 1.34 µM. Both JTE-013 and Fingolimod induced indiscriminate growth inhibition of both cell lines (Figure 2B, Tables S4 and S5). Collectively, these data suggest that inhibition of sphingosine kinase 1, neutral sphingomyelinase, and S1PR1 represent *CDH1^−/−^* cell-specific vulnerabilities.

### 3.3. E-Cadherin-Null Cells Exhibit Vulnerabilities in Clathrin- and Flotillin-Mediated Endocytosis

**Figure 3:**
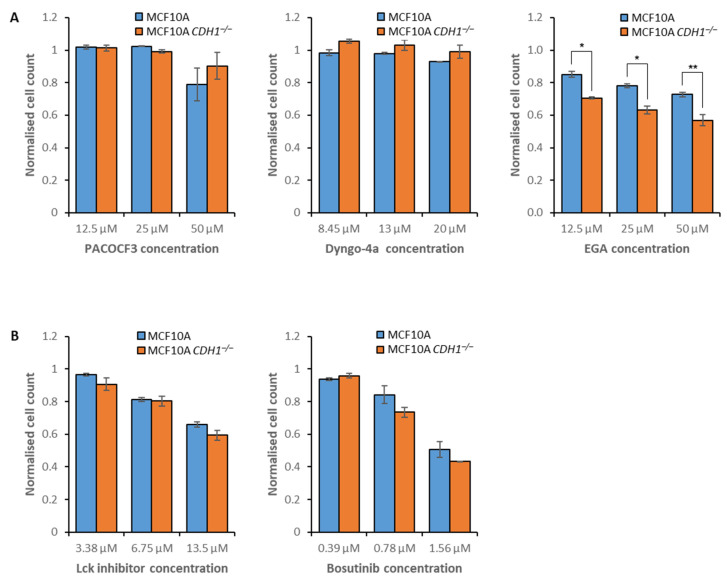
Efficacy of compounds inhibiting endocytosis and Src family kinases. MCF10A and MCF10A *CDH1^−/−^* cells were drugged, then viability was quantified through nuclei counting, and normalization to vehicle controls. Although a nine-point serial dilution was assessed, only the three consecutive compound concentrations that exhibited the greatest difference in viability between *CDH1*^+/+^ and *CDH1^−/−^* cells are depicted. Where no toxicity was observed, the maximum assessed concentrations are depicted. (**A**) Cell viability following treatment with three endocytosis inhibitors. (**B**) Cell viability following treatment with inhibitors of Src family kinases. Average values were calculated from three biological replicates, with +/− 1 standard error of the mean depicted by error bars. *P*-values were calculated using Student’s *t*-test; * *p* ≤ 0.05, ** *p* ≤ 0.01.

Inhibitors of vesicle formation (MNS), clathrin- (chlorpromazine) and flotillin-mediated endocytosis (PP1, PP2 and SU6656) were capable of preferentially inhibiting MCF10A *CDH1^−/−^* cell growth (Figure 1). To further explore endocytosis as a *CDH1^−/−^* cell-specific vulnerability, three additional inhibitors were assessed in MCF10A cells: Dyngo-4a, PACOCF3, and EGA. Dyngo-4a disrupts dynamin-mediated endocytosis, and PACOCF3 inhibits phospholipase A_2_, which is involved in the trafficking of several ligands and receptors across different endocytosis pathways [59]. However, no decrease in either *CDH1*^+/+^ or *CDH1^−/−^* cell numbers was observed following Dyngo-4a treatment, and PACOCF3 inhibited both cell lines indiscriminately (Figure 3A, Tables S6 and S7). Although the precise mechanism is unknown, EGA inhibits the maturation of late endosomes [60] and was more toxic to *CDH1^−/−^* cells across several concentrations (Figure 3A, Tables S6 and S7). In contrast to the MCF10A data, NCI-N87 *CDH1^−/−^* cells were less sensitive to PP1 than NCI-N87 *CDH1^+/+^* cells (Figure S2B, Tables S12 and S13). However, concordant with the MCF10A results, NCI-N87 *CDH1^−/−^* cells were more sensitive to treatment with chlorpromazine than *CDH1*^+/+^ cells (Figure S2C, Tables S12 and S13).

The Src family kinase inhibitors PP1, PP2 and SU6656 were initially selected for their ability to disrupt flotillin-mediated endocytosis through Fyn kinase inhibition. However, all three compounds also inhibit other Src family kinases, including Lck, Hck, c-Src, Yes, and Lyn (Table S8). To determine whether the inhibition of other Src family kinases was responsible for the synthetic lethal phenotype, we assessed two additional inhibitors that are highly specific for individual Src family kinases: the c-Src inhibitor bosutinib and Lck inhibitor. Neither drug caused a synthetic lethal effect in the MCF10A isogenic cell lines, although *CDH1^−/−^* cells displayed a small, non-significant increase in sensitivity to bosutinib (Figure 3B, Tables S6 and S7). Since the only other target shared between PP1, PP2, and SU6656 is Fyn kinase, we hypothesize that inhibition of this kinase, or combined inhibition of Fyn kinase and c-Src, is required for the synthetic lethal phenotype we have observed with Src family kinases.

### 3.4. Disruption of Autophagy Preferentially Inhibits the Growth of Non-Tumorigenic CDH1^−/−^ Cells

**Figure 4:**
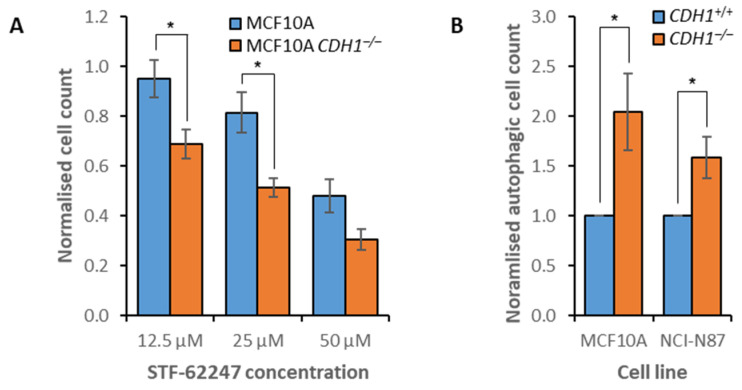
Differences in autophagy between *CDH1*^+/+^ and *CDH1^−/−^* cells. (**A**) MCF10A and MCF10A *CDH1^−/−^* cells were drugged with STF-62247, then viability was quantified through nuclei counting, and normalization to vehicle controls. Although a nine-point serial dilution was assessed, only the three consecutive compound concentrations that exhibited the greatest difference in viability between *CDH1*^+/+^ and *CDH1^−/−^* cells are depicted. (**B**) MCF10A and NCI-N87 isogenic cell lines were treated with 0.2% DMSO, then autophagic vacuoles were stained and quantified. Average values were calculated from three (MCF10A) or five (NCI-N87) biological replicates. Error bars depict +/− 1 standard error of the mean. *P*-values were calculated using Student’s *t*-test; * *p* ≤ 0.05.

Two potent autophagy inhibitors, chloroquine and hydroxy-chloroquine, showed a synthetic lethal effect in the primary drug screen (Figure 1). Chloroquine accumulates in lysosomes, inhibiting neoglycolipid metabolism and proteolysis, thus preventing degradation of autolysosomes [33]. Hydroxy-chloroquine is a derivative of chloroquine, and inhibits autophagy via the neutralization of lysosomes [34]. However, the specific protein targets of both compounds are unknown. One additional autophagy inhibitor, STF-62247, was assessed to provide further support for autophagy as a defective process in MCF10A *CDH1^−/−^* cells. STF-62247 disrupts lysosome function, although the specific target is unknown. This compound accumulates in lysosomes, and both impairs degradation and causes swelling, resulting in a buildup of large static lysosomes [61]. In agreement with the observed chloroquine and hydroxy-chloroquine inhibition, MCF10A *CDH1^−/−^* cells were more sensitive to STF-62247 treatment than MCF10A *CDH1*^+/+^ cells across several concentrations (Figure 4A, Tables S9 and S10), suggesting that these cells are sensitive to inhibition of autolysosome maturation. To examine the functionality of autophagy in *CDH1*-null cell lines directly, we used an autophagy assay to demonstrate that both MCF10A and NCI-N87 cells upregulated autophagy following *CDH1* loss (Figure 4B). Therefore, increased reliance on autophagy in *CDH1*-null cells may explain the susceptibility of MCF10A *CDH1^−/−^* cells to inhibition by chloroquine, hydroxy-chloroquine, and STF-62247. Surprisingly, both *CDH1*^+/+^ and *CDH1^−/−^* cells derived from the NCI-N87 gastric cancer cell line exhibited similar sensitivity to chloroquine treatment, demonstrating the importance of genetic background to drug response (Figure S1D, Tables S12 and S13).

### 3.5. Combination Drug Treatment Enhances Efficacy against MCF10A CDH1^−/−^ Cells

**Figure 5:**
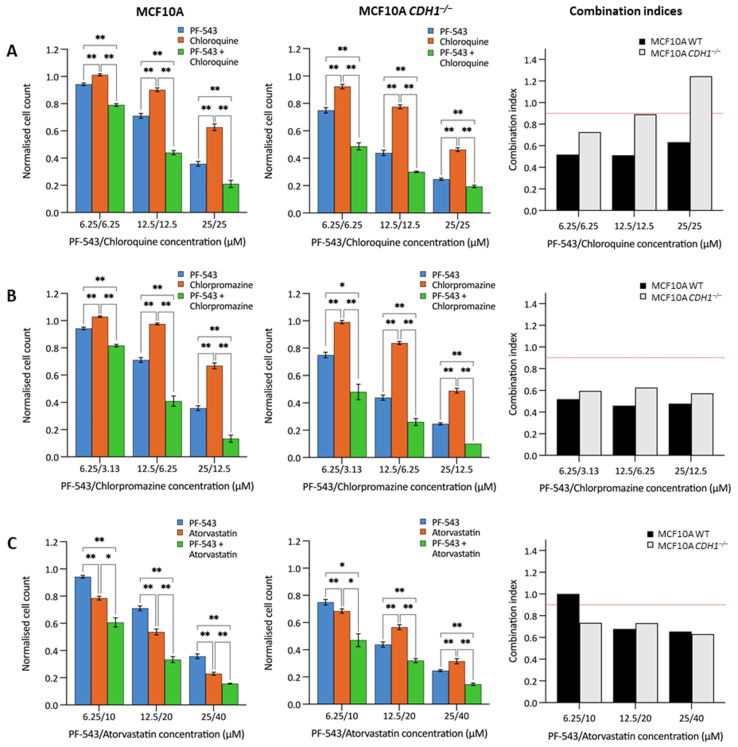
Efficacy of candidate compound combinations. MCF10A (left) and MCF10A *CDH1^−/−^* (middle) cells were drugged with P5-543 in combination with (**A**) chloroquine, (**B**) chlorpromazine, or (**C**) atorvastatin, then viability was quantified through nuclei counting, and normalization to vehicle controls. Treatment with single inhibitors is also depicted. Although an 8-point serial dilution was assessed, only the three greatest concentrations are shown. Combination index values (CI, right) indicate drug interactions. Synergistic effects are represented by CI > 0.9 (depicted by dotted red line), additive effects by CI values between 0.9 and 1.1, and antagonistic effects by CI < 1.1. Error bars depict +/− 1 standard error of the mean. *P*-values were calculated using Student’s *t*-test; * *p* ≤ 0.05, ** *p* ≤ 0.01.

In order to identify synergistic drug combinations, a selection of candidate synthetic lethal compounds were tested together across an 8-point dilution series. Because of its strong synthetic lethal effect, PF-543 was used in each combination, along with three FDA-approved drugs-chloroquine, chlorpromazine and atorvastatin, an inhibitor of cholesterol synthesis that we have previously shown to be synthetic lethal with *CDH1* [15]. Each of these combinations was synergistic in both MCF10A and MCF10A *CDH1^−/−^* cells across most of the tested concentrations (Figure 5, Table S11) [62]. These data suggest that combining synthetic lethal drugs can improve efficacy against *CDH1^−/−^* cells and may enable reduction of the drug dose used for chemoprevention, leading to lower toxicity and greater patient compliance. Additionally, these results highlight sphingosine kinase 1 inhibition as an area of interest for future drug development.

### 3.6. Establishment of a Murine-Derived Gastric Organoid Model of HDGC

**Figure 6:**
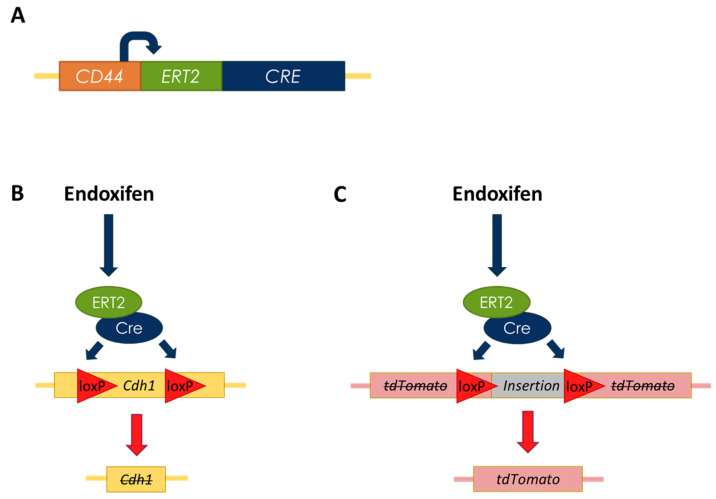
Schematic of transgenic mouse constructs. (**A**) The *CD44* promoter drives expression of a Cre recombinase-ERT2 fusion protein. (**B**) Endoxifen treatment causes the fusion protein to localise to the nucleus. Cre recombinase recognises two loxP sites flanking exons 6 to 10 of the *Cdh1* gene, and excises this region through Cre-mediated recombination, effectively deactivating *Cdh1*. (**C**) Induction of the fusion protein also excises a premature stop codon inserted into a tdTomato construct, inducing expression of the red fluorescent protein tdTomato.

To test the preferred candidate chemoprevention drugs in a more complex model of HDGC, we established gastric organoids from *CD44*-Cre/*Cdh1*^fl/fl^/tdTomato mice (hereafter referred to as *Cdh1*^fl/fl^ organoids). Following the addition of endoxifen to organoid cultures, Cre recombinase activity is induced, resulting in excision of exons 6 to 10 of the *Cdh1* gene, abrogating E-cadherin activity. In addition, a premature stop codon is removed from the tdTomato construct, enabling expression of the red fluorescent protein tdTomato (Figure 6). Organoids were also generated from *CD44*-Cre/tdTomato mice, enabling an E-cadherin-positive control that can be treated with endoxifen to activate tdTomato expression (hereafter referred to as *Cdh1*^+/+^ organoids).

**Figure 7:**
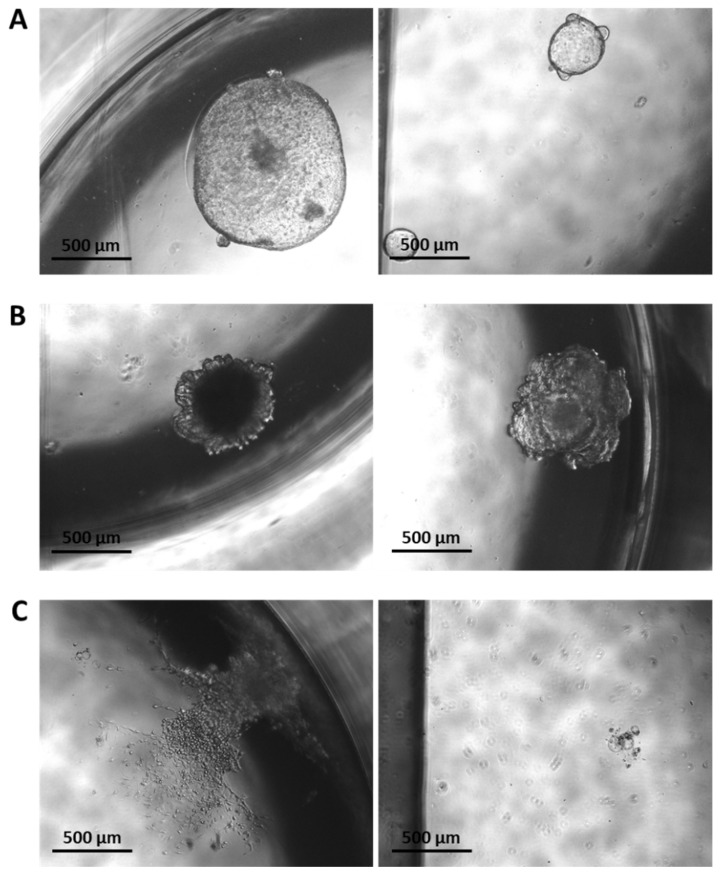
Brightfield microscopy of organoids grown from single tdTomato-positive cells for 11 days. (**A**) Cystic organoids grown from single *Cdh1*^+/+^ cells. (**B**) Organoids grown from single *Cdh1^−/−^* cells with a relatively disrupted structure. (**C**) Most single *Cdh1^−/−^* cells failed to generate organoids, instead presenting with mesenchymal-like (left) or signet ring cell-like (right) morphologies.

*Cdh1*^fl/fl^ and *Cdh1*^+/+^ organoids were induced with endoxifen and disrupted into single-cell suspensions, and then single tdTomato-positive cells were seeded following fluorescence-activated cell sorting. After a period of 11 days, single *Cdh1*^+/+^ cells grew into cystic gastric organoids (Figure 7A). However, only a small number of single *Cdh1^−/−^* cells were capable of generating organoids, and these exhibited both a disorganized structure and lacked a transparent lumen (Figure 7B). Most *Cdh1^−/−^* cells instead grew as a 2-dimensional layer of cells within the Matrigel (Figure 7C). Some of these cells presented with an elongated morphology and decreased cell–cell contacts, both characteristic of mesenchymal cells. Additionally, some cells presented with a swollen cytoplasm, similar in morphology to signet ring cells, which are the typical constituents of early HDGC lesions. Although interesting findings, potentially representing both active epithelial–mesenchymal transition (EMT) signaling and signet ring cell formation, the failure of single *Cdh1^−/−^* cells to generate organoids necessitated an alternative approach to generate 3-dimensional structures suitable for comparison with *Cdh1*^+/+^ organoids.

**Figure 8:**
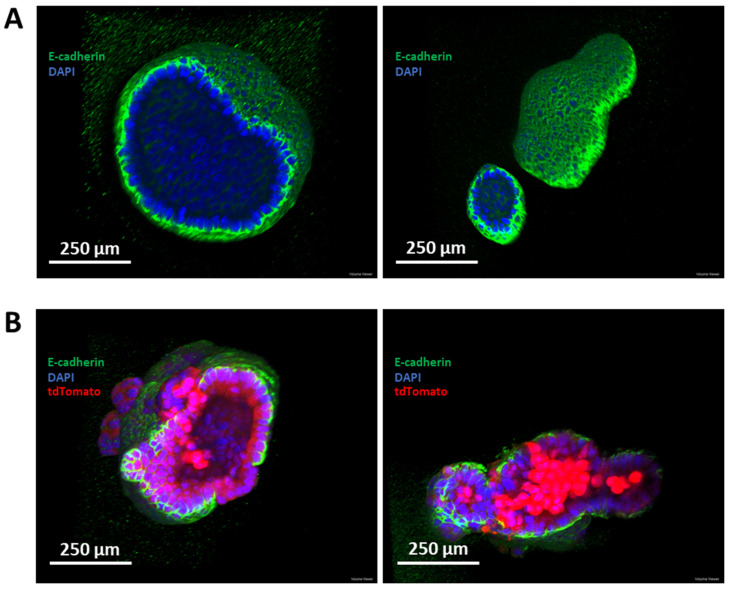
Confocal microscopy of organoids. Immunofluorescence was performed against E-cadherin, with DAPI-stained nuclei. Confocal images represent the focal planes running through the approximate centre of the organoids. (**A**) Highly structured organoids derived from *Cdh1*^fl/fl^ mice without induction of Cre recombinase. (**B**) Relatively disorganised organoids derived from *Cdh1*^fl/fl^ mice with induction of Cre recombinase. Some organoids maintain a hollow lumen (left), while others contain a dense core of *Cdh1^−/−^* cells.

It was hypothesized that, if given an initial period of growth without endoxifen treatment, *Cdh1*^fl/fl^ organoids would generate a sufficient structure to prevent *Cdh1* loss completely abrogating 3-dimensional organization. *Cdh1*^fl/fl^ organoids were grown for 24 h, then treated with endoxifen to activate Cre recombinase, or treated with a DMSO vehicle control. After a further 72 h of growth, immunofluorescence was performed against E-cadherin, and confocal microscopy was utilized to assess the 3-dimensional structure. DMSO-treated organoids presented a highly organized structure consisting of a monolayer of E-cadherin-positive cells surrounding a hollow lumen (Figure 8A). Endoxifen-treated *Cdh1*^fl/fl^ organoids were capable of maintaining a 3-dimensional structure, in contrast to the results from single *Cdh1^−/−^* cells. These organoids displayed a relatively disorganized structure, with clusters of *Cdh1^−/−^/*tdTomato-positive cells expanding as small lesions outside of the epithelial plane (Figure 8B). Some organoids maintained a hollow lumen, while others contained a dense core of *Cdh1^−/−^* cells.

### 3.7. Validation of Candidate Synthetic Lethal Compounds in Organoid Models of HDGC

To validate the candidate synthetic lethal pathways identified during MCF10A drug screening, a single inhibitor of each of sphingolipid signaling (PF-543), autophagy (chloroquine), clathrin- (chlorpromazine), and flotillin-mediated endocytosis (PP1) was selected. A 3-point serial dilution of each drug was assessed in both the gastric organoid model described here, and a murine-derived mammary organoid model also established from *CD44*-Cre/tdTomato and *CD44*-Cre/*Cdh1*^fl/fl^/tdTomato mice (manuscript in preparation).

**Figure 9:**
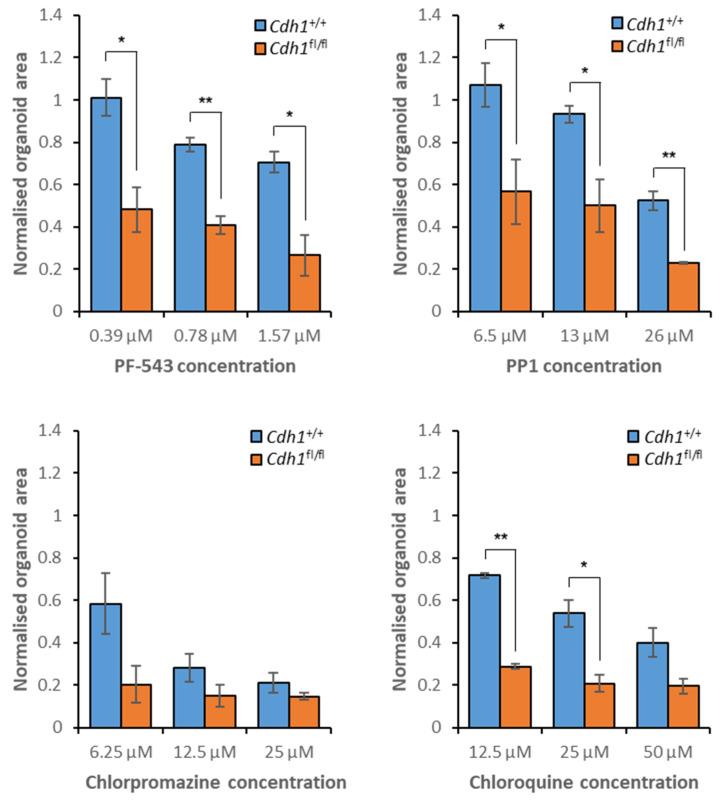
Sensitivity of gastric organoids to treatment with candidate synthetic lethal compounds. Both *Cdh1*^+/+^ and *Cdh1*^fl/fl^ organoids were drugged, then viability was quantified through measurement of organoid area, normalized to vehicle controls. Average values were calculated from three biological replicates, with +/- 1 standard error of the mean depicted by error bars. *P*-values were calculated using Student’s *t*-test; * *p* ≤ 0.05, ** *p* ≤ 0.01.

In the gastric organoid model of HDGC, both the sphingosine kinase 1 inhibitor PF-543 and the c-Src/Fyn kinase inhibitor PP1 preferentially inhibited the growth of *Cdh1*^fl/fl^ organoids across all assessed concentrations (Figure 9, Tables S14 and S15). Chlorpromazine induced a synthetic lethal effect at 6.25 µM but was highly toxic to both organoids at greater concentrations (Figure 9, Tables S14 and S15). Chloroquine induced a synthetic lethal effect at both 12.5 µM and 25 µM but was also highly toxic regardless of *Cdh1* status at 50 µM (Figure 9, Tables S14 and S15).

**Figure 10:**
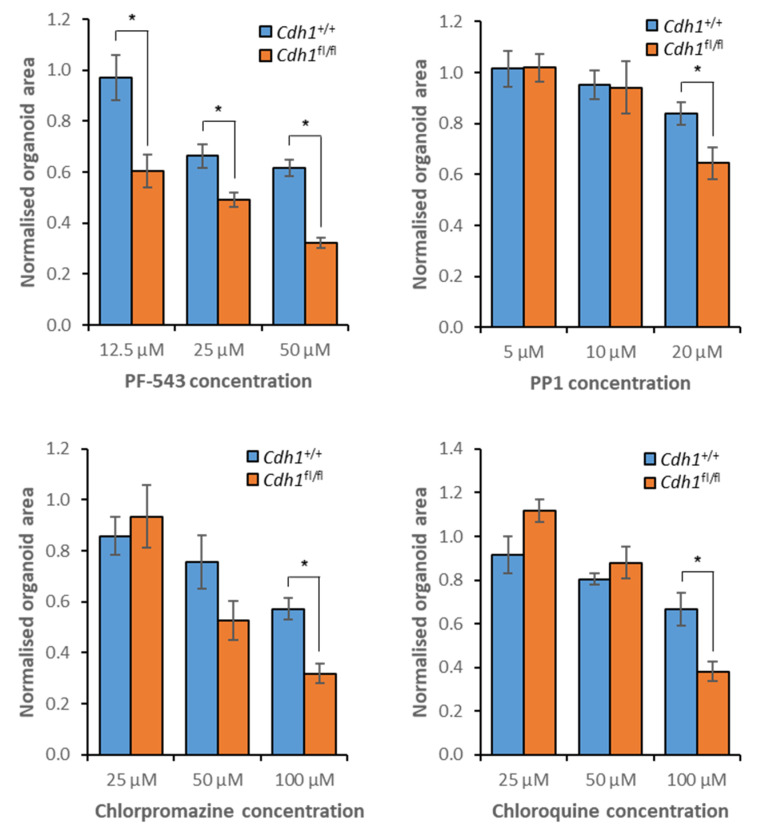
Sensitivity of mammary organoids to treatment with candidate synthetic lethal compounds. Both *Cdh1*^+/+^ and *Cdh1*^fl/fl^ organoids were drugged, then viability was quantified through measurement of organoid area, normalized to vehicle controls. Average values were calculated from three biological replicates, with +/− 1 standard error of the mean depicted by error bars. *P*-values were calculated using Student’s *t*-test; * *p* ≤ 0.05.

When mammary organoids were treated with PF-543, synthetic lethality was observed across all assessed concentrations (Figure 10, Tables S16 and S17). Chlorpromazine induced a synthetic lethal effect at both 50 µM and 100 µM, but synthetic lethality was only observed for PP1 and chloroquine at elevated concentrations (Figure 10, Tables S16 and S17).

Taken together, the drug screening data from MCF10A cells, gastric organoids, and mammary organoids demonstrate the vulnerability of *CDH1*-null cells to inhibition of sphingolipid signaling, autophagy, and endocytosis.

## 4. Discussion

In this study, we have utilized an MCF10A model of HDGC to identify novel druggable vulnerabilities in *CDH1^−/−^* cells. Inhibition of sphingolipid metabolism, autophagy, clathrin-, and flotillin-mediated endocytosis was capable of preferentially inhibiting *CDH1^−/−^* cell growth, and the single most promising inhibitor from each pathway was validated in both gastric and mammary organoid models of HDGC.

Although inhibition of *de novo* sphingolipid synthesis was not synthetic lethal with *CDH1*, the inhibition of neutral sphingomyelinase with GW4869, responsible for converting membrane-associated sphingomyelin into ceramide [50], preferentially inhibited MCF10A *CDH1^−/−^* cell growth. Sphingomyelin represents the primary source of sphingolipids within the plasma membrane [50] and the only source of ceramide. Ceramide is required for the translocation of some proteins across lipid raft boundaries and is required for the formation of ceramide-rich platforms, hypothesized to be important in the clustering of specific receptors [63]. The cortical actin cytoskeleton is a critical component of lipid raft domain homeostasis [63], and thus the disorganized actin cytoskeleton in *CDH1^−/−^* cells is predicted to result in poorly maintained membrane compartmentalization. Treatment with GW4869, which has previously been shown to induce lipid raft defects and reduce protein association with these regions [63], may further disrupt the organization of these rafts in *CDH1^−/−^* cells. This would result in impaired membrane trafficking regulation and perturbation of stable signaling hubs while leaving *CDH1*^+/+^ cells relatively unharmed. Supporting this, our laboratory has previously found that depletion of membrane-associated cholesterol, a critical component of lipid raft organization [64], through treatment with statins [13,19] or methyl-β-cyclodextrin is synthetic lethal [15]. Alternatively, ceramide depletion may instead deprive cells of sphingosine, which ceramidase can generate from ceramide [65].

Membrane-associated sphingosine can be phosphorylated by sphingosine kinase 1 to generate sphingosine-1-phosphate, a bioactive sphingolipid. Sphingosine-1-phosphate can be secreted and subsequently binds to S1PRs 1–5, activating downstream signaling dependent on the bound receptor. Inhibition of both sphingosine kinase 1 and S1PR1 induced a synthetic lethal effect. However, sphingosine kinase 1 inhibition was more potent. These data suggest that either other functions of sphingosine-1-phosphate are vulnerable to E-cadherin loss or another S1PR is responsible for part of the synthetic lethal mechanism. Considering the low solubility of the only currently available S1PR3 inhibitor VPC 23019, S1PR3 cannot be ruled out as a potential synthetic lethal candidate. S1PR1 and S1PR3 are localized to lipid rafts regions, whereas S1PR2 is found both within raft regions and dispersed throughout the membrane [66]. Additionally, normal sphingosine kinase 1 function requires lipid raft association [67], and at least one sphingosine-1-phosphate transporter, required for extracellular secretion, is localized to lipid rafts [66]. The hypothesized inability of *CDH1^−/−^* cells to effectively maintain lipid rafts is predicted to result in defective sphingosine kinase 1 activity, sphingosine-1-phosphate secretion, and clustering of both S1PR1 and S1PR3, thus reducing the activation of downstream signaling. Further disruption of this signaling through inhibition of S1PRs or sphingosine-1-phosphate generation is predicted to result in *CDH1^−/−^* cell-specific growth inhibition or death.

Inhibition of endocytosis mechanisms that are shared by several endocytic pathways exhibited mixed synthetic lethality with *CDH1*. Treatment with inhibitors of dynamin or phospholipase A_2_ were not synthetic lethal, whereas inhibitors of endosome generation and maturation induced a synthetic lethal effect. In contrast to these mixed results, the specific inhibition of clathrin- and flotillin-mediated endocytic pathways induced preferential inhibition of MCF10A *CDH1^−/−^* cell growth.

Although only one of three clathrin-mediated endocytosis inhibitors were synthetic lethal, both concanavalin A and phenylarsine oxide have been shown to interfere with G protein-coupled receptor signaling [24] and both micropinocytosis and phagocytosis, respectively [68,69]. However, the only currently available clathrin-endocytosis specific inhibitor, chlorpromazine, was effective at preferentially inhibiting MCF10A *CDH1^−/−^* and NCI-N87 *CDH1^−/−^* cell growth. The invagination of clathrin-coated pits is actin-dependent [70], as well as the encapsulation of the immature vesicle by actin filaments [71]. Additionally, homeostasis of plasma membrane tension is primarily regulated through actin filament-plasma membrane interactions [72], and this tension has a direct effect on clathrin-mediated endocytosis. If tension maintenance is disrupted, clathrin polymerization is halted, and endocytosis inhibited [73]. Consistent with this, the disruption of actin dynamics results in clathrin-mediated endocytosis inhibition [74], and it is predicted that the disorganized cell cytoskeleton in *CDH1^−/−^* cells induces similar effects. Partially perturbed clathrin-mediated endocytosis is hypothesized to result in deficits in the ability of *CDH1^−/−^* cells to internalize nutrients, regulate crucial intercellular signaling pathways, and regulate receptor recycling [75]. Further disruption of this vulnerability via chlorpromazine treatment may breach a critical functional threshold, resulting in *CDH1^−/−^* cell-specific growth inhibition.

Three inhibitors of flotillin-mediated endocytosis (PP1, PP2 and SU6656) were identified as promising synthetic lethal candidate compounds. Each of these compounds inhibits additional Src family kinases but share the targets Fyn kinase and c-Src. Fyn kinase is a critical component of flotillin-mediated endocytosis, a process requiring lipid raft stabilization and organization, and as described above, this is hypothesized to be perturbed in *CDH1^−/−^* cells. By extension, flotillin-mediated endocytosis is likely defective, potentially due to interruption of the organization and co-localization of flotillin-1 and -2 in sufficient levels to initiate membrane invagination [76]. Additionally, flotillin-mediated endocytosis can be triggered by a sufficient concentration of glycosylphosphatidylinositol-anchored proteins, and this localization is reliant upon F-actin dynamics to stabilize their positioning [77,78]. Flotillin-mediated endocytosis is dependent upon actin filament organization and polymerization for vesicle scission from the membrane and subsequent reorganization of the local membrane region to enable the generation of early endosomes [79,80]. A combination of these effects is predicted to decrease the efficiency of flotillin-mediated endocytosis in *CDH1^−/−^* cells, resulting in a vulnerability to further disruption. Similar to clathrin-mediated endocytosis, this likely restricts the ability of cells to internalize important cargo and recycle crucial receptors, thus preferentially inhibiting the growth of *CDH1^−/−^* cells. It is important to note that Fyn kinase is also involved in several other biological pathways, including cell survival, proliferation, adhesion, and invasion [81,82,83]. Therefore, it is possible that these pathways are at least partially responsible for the observed synthetic lethal phenotype. Unfortunately, no specific inhibitors of either Fyn kinase or flotillin-mediated endocytosis are currently available, precluding confirmation of this via a drug screening approach. However, combined with our recent finding that *CDH1^−/−^* cells present with decreased endocytosis [15], and a vulnerability in clathrin-mediated endocytosis, flotillin-mediated endocytosis is proposed as the mechanism underlying the sensitivity to PP1, PP2 and SU6656 treatment.

Inhibition of autophagy through treatment with three compounds (chloroquine, hydroxy-chloroquine and STF-62247) was synthetic lethal with *CDH1*. Although the specific protein targets of each compound are unknown, all three share a common mechanism of impairing autolysosomes [33,34,84], the final vesicle that degrades autophagic cargo for nutrient release [84]. Actin plays a crucial role in initiating the synthesis of the autophagosome, the vesicle that encapsulates autophagic cargo destined for degradation [85,86]. Actin both recruits protein complexes required for synthesis and provides scaffolding for vesicle assembly [85,86]. As a result, when actin dynamics are disrupted, such as in *CDH1^−/−^* cells, autophagosome synthesis is likely to be perturbed. Following synthesis, the autophagosome is transported along microtubule networks for lysosome fusion, resulting in autolysosome formation [84]. If this microtubule network is already disrupted due to *CDH1* loss, this transport is likely to be perturbed or delayed, decreasing the overall rate of autophagy. Autophagic lysosomes are recycled through the extrusion of tubules and vesicles from a lysosome, which then interact to generate a fresh lysosome [87]. In *CDH1^−/−^* cells, the disorganized microtubule network may reduce the ability of cells to direct this tubule–vesicle interaction, impairing lysosomal recycling. This would result in an autophagy bottleneck, with insufficient lysosomes for autolysosome generation. A similar model has been described in VHL-deficient cells, which cannot recycle lysosomes [88]. When treated with STF-62247, one of the autophagy inhibitors assessed in this study, VHL-deficient cells cannot survive the accumulation of swollen autolysosomal structures resulting from treatment, whereas VHL-positive cells can survive treatment, hypothesized to result from their ability to efficiently recycle lysosomes, thus having less reliance upon the degradation of the swollen autolysosomes [61]. Additionally, we have observed an upregulation of autophagy in both MCF10A and NCI-N87 cells following the loss of *CDH1*. Autophagy upregulation may enable *CDH1^−/−^* cells to overcome these autophagy bottlenecks or to compensate for deficits in nutrient uptake resulting from defective endocytosis. A combination of these autophagy defects is proposed to result in *CDH1^−/−^* cell-specific vulnerabilities to further inhibition.

It should be emphasized that compounds commonly exhibit off-target effects, and the targets of inhibitors often function across multiple biological pathways. In addition, although each pathway of interest has been described in this study as distinct, the different forms of vesicle trafficking and sphingolipid metabolism are complex, interlinked biological processes. For example, autophagy can be regulated through sphingosine kinase activity [89], which can be internalized through clathrin-mediated endocytosis [90]. Flotillins can also directly interact with and regulate local sphingosine levels within lipid rafts [91], and they rely upon specific compositions of sphingolipids to enable flotillin-mediated endocytosis [92]. These combined complexities make it difficult to determine the definitive underlying mechanism of synthetic lethality with a drug screening approach, and further investigation through the application of siRNA or CRISPR/Cas9 screening, or functional assays on pathways of interest, would help to validate these findings. However, drug screening has the advantage of a clear pathway to clinical utility. Although our arbitrary threshold for synthetic lethality, requiring a difference in cell viability of >15%, is relatively low, it is based on a short 48 h treatment period. We anticipate the effects at the lower end of the tested concentration range will be more pronounced following extended treatment *in vivo*. Regardless, these effects identify pathways or mechanisms that are intrinsically linked to E-cadherin function, providing a basis for further investigation and possible novel, synergistic combinations.

This study has utilized nuclei counting for viability quantification, and although this approach is effective at determining growth inhibition, cell death has not been confirmed. Chloroquine, chlorpromazine, PP1, and PF-543 have all been shown to induce apoptosis in other biological systems [93,94]. However, this will be confirmed in our models of HDGC prior to advancing into *in vivo* models.

To extend our preclinical HDGC models, we have established a murine-derived gastric organoid model of *Cdh1* loss. The gastric cells comprising these organoids are capable of differentiating into all gastric lineages, aside from parietal cells [95], making this a more complex preclinical model for drug screening. The generation of isogenic organoid cultures with and without functional *Cdh1* was not possible from single cells, with the majority of *Cdh1^−/−^* cultures incapable of expanding into 3-dimensional structures. This is perhaps unsurprising due to E-cadherin’s role in cell–cell adhesion, regulation of tissue tension, and maintaining epithelial cell polarity [96,97,98]. Individual *Cdh1^−/−^* cells commonly exhibited a mesenchymal morphology, potentially indicative of active EMT signaling, but this requires further validation. Additionally, cells similar in appearance to signet ring cells, the typical constituents of HDGC stage T1a lesions [99], were common. When single cells were grown for 24 h prior to Cre recombinase induction, 3-dimensional structures grew from both *Cdh1*^+/+^ and *Cdh1^−/−^* cells. *Cdh1*^fl/fl^ cultures exhibited relatively disorganized structures, presenting with clusters of *Cdh1^−/−^*/tdTomato-positive cells dividing outside of a predominantly *Cdh1*^+/+^ epithelial plane. This observation is consistent with current hypotheses surrounding HDGC initiation, whereby *CDH1^−/−^* cells divide outside of the epithelial plane, escape normal growth signaling regulation, and develop into lesions [100]. In addition, we have established a murine-derived mammary organoid model of *Cdh1* loss (manuscript in preparation) to provide models of lobular breast cancer in HDGC patients. Four candidate synthetic lethal compounds were assessed in each organoid model of HDGC, and all exhibited increased toxicity against *Cdh1*^fl/fl^ organoids.

Although this research has focused on the chemoprevention of HDGC, the resistance of NCI-N87 *CDH1^−/−^* cells, representative of advanced gastric cancer, to all candidate compounds except for chlorpromazine, necessitates further investigation into drug efficacy in advanced cancer backgrounds. We are developing genetically modified mice with additional oncogenic drivers, such as loss of *Trp53*, in order to address this issue in a system more representative of HDGC. Activity in these models may open the way for new approaches for treating various sporadic cancers with E-cadherin deficiency [7,8,9,10], and may reduce the likelihood of early HDGC lesions developing therapy resistance through additional oncogenic events.

Considering that both chloroquine and chlorpromazine are FDA approved, these drugs are particularly interesting candidates for further development as potential HDGC chemoprevention agents. However, to enable long-term use, it will be important to minimize drug side effects. For chlorpromazine, this is likely to require chemical modification or novel formulations to reduce its ability to cross the blood–brain barrier. Chloroquine may inhibit *CDH1*-null cells at concentrations routinely achieved during malaria treatments [101,102]. However, its use as part of a synergistic combination of drugs that lack overlapping toxicities is likely to be required for it to be considered for chemoprevention. Alternatively, the development of a stomach-targeting drug delivery system would provide the means to minimize the systemic side effects of all chemoprevention drugs. Fortunately, given the relatively indolent nature of the early-stage gastric lesions in *CDH1* mutation carriers, extended intervals between repeat drug administrations are likely to be effective, perhaps every 1–3 years.

## 5. Conclusions

By applying a drug screening approach to MCF10A, NCI-N87, gastric organoid and mammary organoid models of E-cadherin loss, this study has identified sphingolipid signaling, endocytosis, and autophagy as promising druggable vulnerabilities in *CDH1^−/−^* cells. With further research, these compounds may lead to the development of novel HDGC chemoprevention strategies, thus potentially offering an alternative to prophylactic total gastrectomy.

## Figures and Tables

**Table 1:** Summary of 26 compounds assessed during the initial drug screen. Compounds are separated by process, and the underlying specific biological pathways.

Process	Pathway of Interest	Compound
Endocytosis	Clathrin-mediated endocytosis	Concanavilin A [24]
		Phenylarsine oxide [25]
		Chlorpromazine [25]
	Flotillin-mediated endocytosis	PP1 [26]
		PP2 [26]
		SU6656 [27]
	Vesicle formation	DBeQ [28,29]
		NMS-873 [29,30]
		3,4-methylenedioxy-β-nitrostyrene (MNS) [28,29]
	Sialic acid-mediated endocytosis	N-Acetyl-2,3-dehydro-2-deoxyneuraminic acid [31]
		Oseltamivir [31]
	Caveolae-mediated endocytosis	Genistein [32]
Autophagy	Endosome acidification	Chloroquine [33]
		Hydroxy-chloroquine [34]
Intracellular vesicle trafficking	Golgi apparatus vesicle transport	Golgicide A [35]
		Brefeldin A [36]
	Endoplasmic reticulum vesicle transport	Cyclosporin [37]
	Nuclear export	Leptomycin B [38]
	Gap junction vesicle transport	18α-glycyrrhetinic acid [39]
		Carbenoxolone [40]
Plasma membrane organisation	Sphingolipid metabolism	Myriocin [41]
		Fumonisin B1 [42]
		ABC294640 [43]
		SKI-11 [44]
		Ponesimod [45]
		PF-543 [46]

## Data Availability

Data is contained within the article or supplementary material.

## Supplementary Materials

The following are available online at https://www.mdpi.com/article/10.3390/cancers14010102/s1, Figure S1: Representative brightfield microscopy images of organoids following treatment with either DMSO or PF-543. Figure S2: Efficacy of candidate synthetic lethal compounds in isogenic NCI-N87 cells. Table S1: List of compounds utilized within this study, the solvents used for reconstitution, and compound suppliers. Table S2: Cell viability ratios from initial drug screening. Table S3: IC_50_ values from initial drug screening. Table S4: Cell viability ratios following inhibition of sphingolipid metabolism and signaling. Table S5: IC_50_ values from inhibition of sphingolipid metabolism and signaling. Table S6: Cell viability ratios following inhibition of endocytosis or Src family kinases. Table S7: IC_50_ values from inhibition of endocytosis or Src family kinases. Table S8: Targets of Src family kinase inhibitors. Targets are ordered in ascending order of IC50 values. Table S9: Cell viability ratios following inhibition of autophagy. Table S10: IC_50_ values from inhibition of autophagy. Table S11: MCF10A cell viability ratios following treatment with drug combinations. Table S12: NCI-N87 cell viability ratios following treatment with candidate synthetic lethal compounds. Table S13: IC_50_ values for candidate synthetic lethal compounds in NCI-N87 isogenic cells. Table S14: Gastric organoid viability ratios following treatment with candidate synthetic lethal compounds. Table S15: IC_50_ values for candidate synthetic lethal compounds in *Cdh1*^+/+^ and *Cdh1*^fl/fl^ gastric organoids. Table S16: Mammary organoid viability ratios following treatment with candidate synthetic lethal compounds. Table S17: IC_50_ values for candidate synthetic lethal compounds in *Cdh1*^+/+^ and *Cdh1*^fl/fl^ mammary organoids.
