# Supplementary material for: Loss of E-Cadherin Leads to Druggable Vulnerabilities in Sphingolipid Metabolism and Vesicle Trafficking
Source: Cancers (Basel). 2021 Dec 26;14(1):102. doi: 10.3390/cancers14010102 (PMC8749886; doi:10.3390/cancers14010102)
Supplement: Supplementary file 1 [file cancers-14-00102-s001.zip › supplementary.pdf]

cancers

MDPI

**Table S1.** List of compounds utilized within this study, the solvents used for reconstitution, and compound suppliers.

| Compound | Solvent | Supplier |
|---|---|---|
| 18α-glycyrrhetinic acid | Ethanol | Sigma-Aldrich, USA |
| 3,4-Methylenedioxy-β-nitrostyrene | DMSO | Sapphire North America, USA |
| ABC294640 | DMSO | Selleckchem, USA |
| Atorvastatin | DMSO | Sapphire North America, USA |
| Bosutinib | DMSO | Cayman Chemical, USA |
| Brefeldin A | DMSO | Selleckchem, USA |
| Carbenoxolone | Milli-Q $H_2O$ | Tocris Bioscience, UK |
| Chloroquine | Milli-Q $H_2O$ | Selleckchem, USA |
| Chlorpromazine | DMSO | Selleckchem, USA |
| Concanavilin A | PBS | Sigma-Aldrich, USA |
| Cyclosporin | DMSO | Selleckchem, USA |
| DBeQ | DMSO | Selleckchem, USA |
| Dyngo-4a | DMSO | Selleckchem, USA |
| EGA | DMSO | Sigma-Aldrich, USA |
| Fingolimod | DMSO | Cayman Chemical, USA |
| Fumonisin B1 | DMSO | Sigma-Aldrich, USA |
| Genistein | DMSO | Selleckchem, USA |
| Golgicide A | DMSO | Selleckchem, USA |
| GW4869 | DMSO | Cayman Chemical, USA |
| Hydroxy-chloroquine | Milli-Q $H_2O$ | Selleckchem, USA |
| JTE-013 | DMSO | Cayman Chemical, USA |
| L-cycloserine | DMSO | Selleckchem, USA |
| Lck inhibitor | DMSO | Cayman Chemical, USA |
| Leptomycin B | Ethanol | Tocris Bioscience, UK |
| MP A08 | DMSO | Cayman Chemical, USA |
| Myriocin | DMSO | Sigma-Aldrich, USA |
| N-Acetyl-2,3-dehydro-2-deoxyneuraminic acid | Milli-Q $H_2O$ | Sigma-Aldrich, USA |
| NMS-873 | DMSO | Selleckchem, USA |
| Oseltamivir | DMSO | Selleckchem, USA |
| PACOCF3 | DMSO | Tocris Bioscience, UK |
| PF-543 | DMSO | Selleckchem, USA |
| Phenylarsine oxide | DMSO | Sigma-Aldrich, USA |
| Ponesimod | DMSO | Selleckchem, USA |
| PP1 | DMSO | Selleckchem, USA |
| PP2 | DMSO | Selleckchem, USA |
| SKI-11 | DMSO | Selleckchem, USA |
| SKI 178 | DMSO | Cayman Chemical, USA |
| SLC5111312 | DMSO | Cayman Chemical, USA |
| STF-62247 | DMSO | Cayman Chemical, USA |
| SU6656 | DMSO | Selleckchem, USA |
| VPC 23019 | DMSO | Cayman Chemical, USA |
| W123 | DMSO | Cayman Chemical, USA |

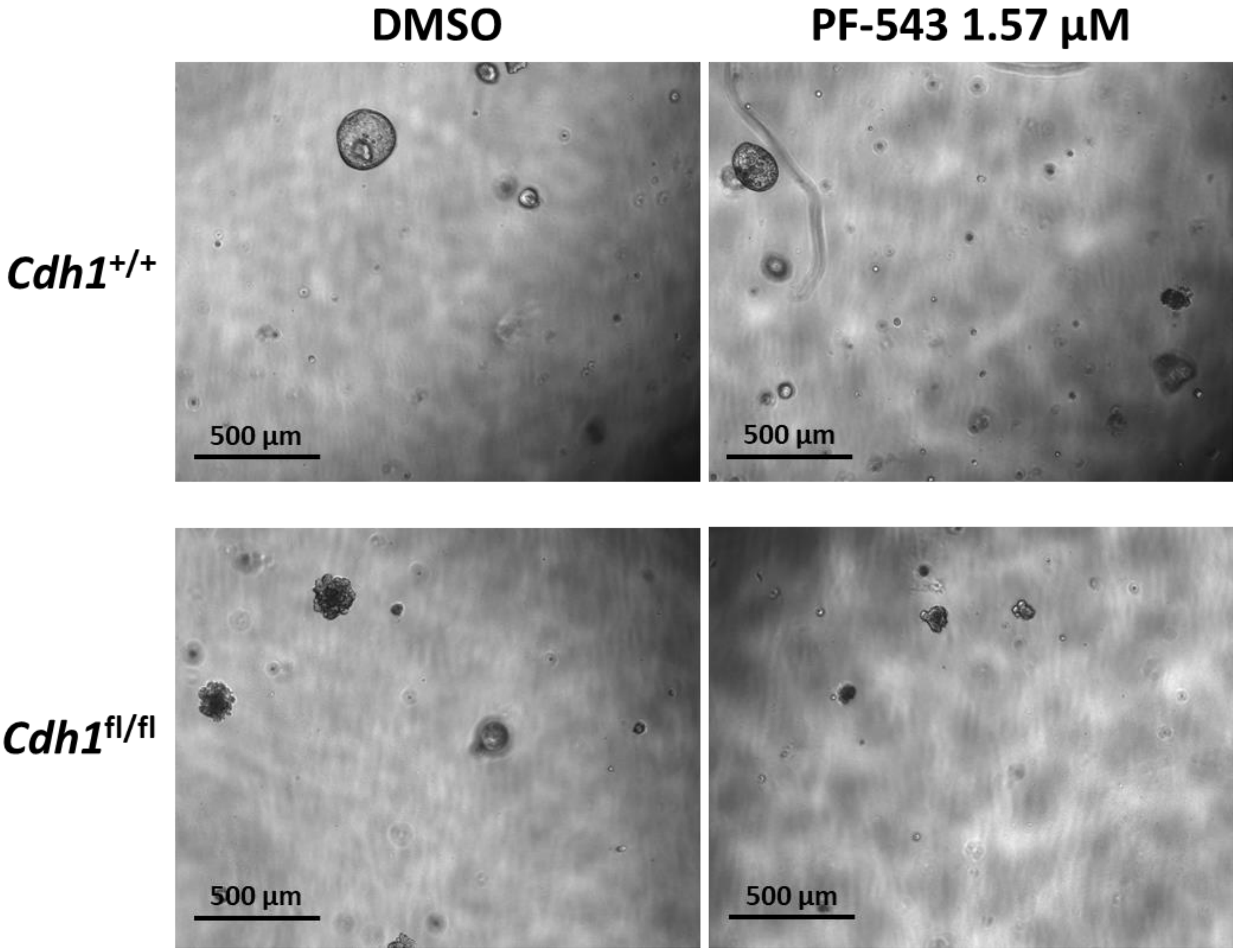


**Figure S1.** Representative brightfield microscopy images of organoids following treatment with either DMSO or PF-543. Multiple images were captured across the surface area of each well, and across multiple focal planes.

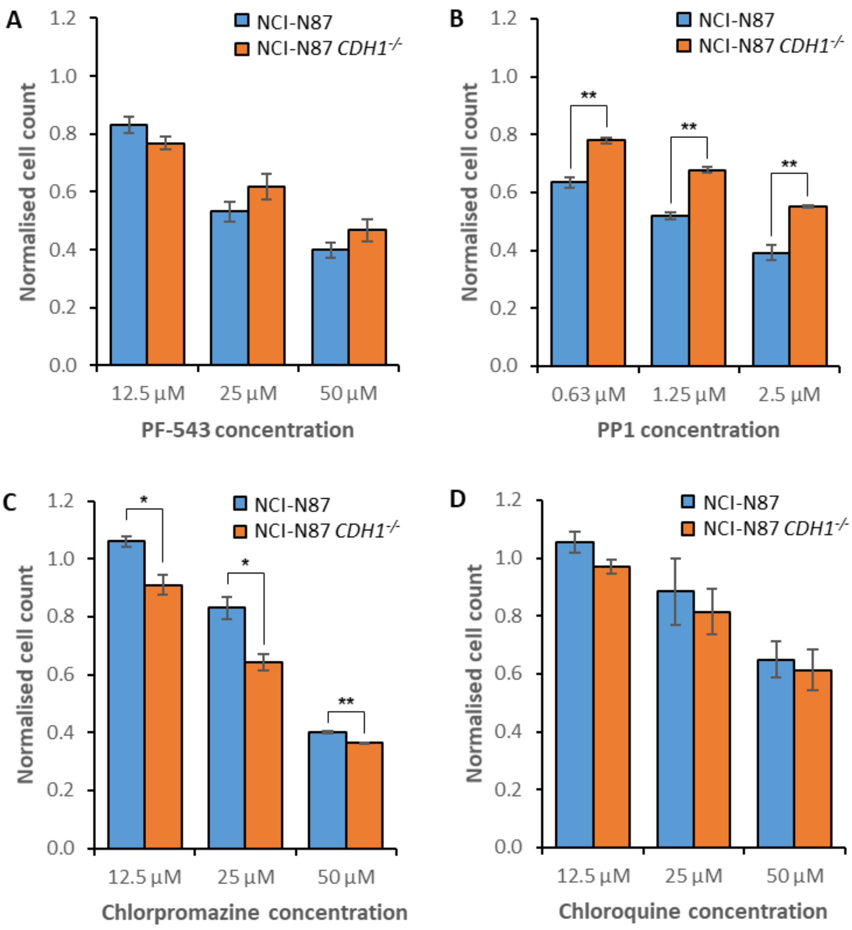


**Figure S2.** Efficacy of candidate synthetic lethal compounds in isogenic NCI-N87 cells. NCI-N87 and NCI-N87 *CDH1*$^{-/-}$ cells were drugged with inhibitors of **(A)** sphingolipid metabolism, **(B)** clathrin-mediated endocytosis, **(C)** flotillin-mediated endocytosis or **(D)** autophagy. Viability was quantified through nuclei counting, and normalisation to vehicle controls. Although a nine-point serial dilution was assessed, only the three consecutive compound concentrations that exhibited the greatest difference in viability between *CDH1*$^{+/+}$ and *CDH1*$^{-/-}$ cells are depicted. Average values were calculated from three biological replicates, with +/- 1 standard error of the mean depicted by error bars. *P*-values were calculated using Student's t-test; * $P < 0.05$, ** $P < 0.01$.

**Table S2.** Cell viability ratios from initial drug screening.

| Compound | Concentration (μM) | Ratio of MCF10A *CDH1*$^{-/-}$ to MCF10A viability |
|---|---|---|
| PF-543 | 0.2 | 1.00 |
| | 0.39 | 1.00 |
| | 0.78 | 0.92 |
| | 1.56 | 0.90 |
| | 3.13 | 0.97 |
| | 6.25 | 0.92 |
| | 12.5 | 0.68 |
| | 25 | 0.58 |
| | 50 | 0.68 |
| Chlorpromazine | 0.2 | 1.00 |
| | 0.39 | 0.98 |
| | 0.78 | 0.98 |
| | 1.56 | 0.94 |
| | 3.13 | 0.96 |
| | 6.25 | 0.80 |
| | 12.5 | 0.61 |
| | 25 | 0.34 |
| | 50 | 0.40 |
| PP1 | 0.64 | 1.00 |
| | 0.98 | 0.93 |
| | 1.51 | 0.88 |
| | 2.32 | 0.92 |
| | 3.57 | 0.84 |
| | 5.49 | 0.73 |
| | 8.45 | 0.65 |
| | 13 | 0.59 |
| | 20 | 0.53 |
| PP2 | 0.64 | 0.91 |
| | 0.98 | 0.88 |
| | 1.51 | 0.84 |
| | 2.32 | 0.84 |
| | 3.57 | 0.81 |
| | 5.49 | 0.70 |
| | 8.45 | 0.66 |
| | 13 | 0.61 |
| | 20 | 0.59 |
| SU6656 | 1 | 1.04 |
| | 1.33 | 1.02 |
| | 1.78 | 0.94 |
| | 2.37 | 0.96 |
| | 3.16 | 0.84 |
| | 4.22 | 0.74 |
| | 5.63 | 0.75 |
| | 7.5 | 0.79 |
| | 10 | 0.89 |

Table continued on next page…

| **Compound** | **Concentration (μM)** | **Ratio of MCF10A *CDH1*$^{-/-}$ to MCF10A viability** |
|---|---|---|
| 3,4-Methylenedioxy-β-nitrostyrene | 0.9 | 0.98 |
| | 1.2 | 0.97 |
| | 1.6 | 0.95 |
| | 2.14 | 0.99 |
| | 2.85 | 0.87 |
| | 3.8 | 0.71 |
| | 5.06 | 0.62 |
| | 6.75 | 0.52 |
| | 9 | 0.58 |
| Chloroquine | 0.2 | 0.98 |
| | 0.39 | 0.94 |
| | 0.78 | 0.92 |
| | 1.56 | 0.91 |
| | 3.13 | 0.94 |
| | 6.25 | 0.88 |
| | 12.5 | 0.73 |
| | 25 | 0.58 |
| | 50 | 0.62 |
| Hydroxy-chloroquine | 0.2 | 1.02 |
| | 0.39 | 1.02 |
| | 0.78 | 1.07 |
| | 1.56 | 1.06 |
| | 3.13 | 0.93 |
| | 6.25 | 0.94 |
| | 12.5 | 0.83 |
| | 25 | 0.73 |
| | 50 | 0.76 |

**Table S3.** $IC_{50}$ values from initial drug screening.

| **Compound** | **Cell line** | **$IC_{50}$ (μM)** |
|---|---|---|
| PF-543 | MCF10A | 33.92 |
| | MCF10A *CDH1*$^{-/-}$ | 14.46 |
| Chlorpromazine | MCF10A | 9.55 |
| | MCF10A *CDH1*$^{-/-}$ | 5.79 |
| PP1 | MCF10A | ≥50 |
| | MCF10A *CDH1*$^{-/-}$ | 11.16 |
| PP2 | MCF10A | ≥50 |
| | MCF10A *CDH1*$^{-/-}$ | 12.66 |
| SU6656 | MCF10A | 4.81 |
| | MCF10A *CDH1*$^{-/-}$ | 4.19 |
| 3,4-Methylenedioxy-β-nitrostyrene | MCF10A | 5.74 |
| | MCF10A *CDH1*$^{-/-}$ | 4.15 |
| Chloroquine | MCF10A | ≥50 |
| | MCF10A *CDH1*$^{-/-}$ | 21.08 |
| Hydroxy-chloroquine | MCF10A | ≥50 |
| | MCF10A *CDH1*$^{-/-}$ | 35.69 |

**Table S4.** Cell viability ratios following inhibition of sphingolipid metabolism and signaling.

| Compound | Concentration (μM) | Ratio of MCF10A *CDH1*$^{-/-}$ to MCF10A viability |
|---|---|---|
| SKI 178 | 0.31 | 0.99 |
| | 0.42 | 1.04 |
| | 0.56 | 0.98 |
| | 0.74 | 0.95 |
| | 0.99 | 1.06 |
| | 1.32 | 1.00 |
| | 1.76 | 0.96 |
| | 2.34 | 0.87 |
| | 3.12 | 0.91 |
| MP A08 | 0.18 | 0.97 |
| | 0.36 | 1.01 |
| | 0.72 | 0.99 |
| | 1.44 | 1.04 |
| | 2.89 | 0.95 |
| | 5.78 | 0.97 |
| | 11.55 | 1.02 |
| | 23.1 | 0.96 |
| | 46.2 | 1.20 |
| SLC5111312 | 0.05 | 1.04 |
| | 0.11 | 1.02 |
| | 0.21 | 1.03 |
| | 0.42 | 1.07 |
| | 0.84 | 0.92 |
| | 1.68 | 0.88 |
| | 3.37 | 0.86 |
| | 6.73 | 0.79 |
| | 13.46 | 0.85 |
| L-cycloserine | 0.2 | 1.08 |
| | 0.39 | 0.99 |
| | 0.78 | 1.01 |
| | 1.56 | 1.02 |
| | 3.13 | 1.03 |
| | 6.25 | 1.02 |
| | 12.5 | 0.98 |
| | 25 | 0.97 |
| | 50 | 1.00 |
| GW4869 | 1.51 | 0.92 |
| | 1.67 | 0.89 |
| | 1.86 | 0.86 |
| | 2.07 | 0.84 |
| | 2.3 | 0.88 |
| | 2.55 | 0.84 |
| | 2.84 | 0.84 |
| | 3.15 | 0.75 |
| | 3.5 | 0.77 |

Table continued on next page…

| Compound | Concentration (μM) | Ratio of MCF10A *CDH1*$^{-/-}$ to MCF10A viability |
|---|---|---|
| W123 | 0.2 | 0.98 |
| | 0.39 | 1.04 |
| | 0.78 | 1.01 |
| | 1.56 | 1.00 |
| | 3.13 | 0.94 |
| | 6.25 | 0.89 |
| | 12.5 | 0.84 |
| | 25 | 0.75 |
| | 50 | 0.73 |
| JTE-013 | 3.23 | 1.03 |
| | 4.55 | 1.02 |
| | 6.41 | 1.01 |
| | 9.02 | 1.01 |
| | 12.7 | 1.03 |
| | 17.9 | 0.99 |
| | 25.2 | 0.96 |
| | 35.5 | 0.91 |
| | 50 | 0.94 |
| VPC 23019 | 0.005 | 0.96 |
| | 0.01 | 0.96 |
| | 0.02 | 0.99 |
| | 0.04 | 0.95 |
| | 0.08 | 0.98 |
| | 0.17 | 0.96 |
| | 0.34 | 0.95 |
| | 0.67 | 0.94 |
| | 1.34 | 0.94 |
| Fingolimod | 0.21 | 0.94 |
| | 0.35 | 0.89 |
| | 0.58 | 0.90 |
| | 0.97 | 0.87 |
| | 1.62 | 0.93 |
| | 2.7 | 1.08 |
| | 4.5 | 1.03 |
| | 7.5 | 1.18 |
| | 12.5 | 1.51 |

**Table S5.** $IC_{50}$ values from inhibition of sphingolipid metabolism and signaling.

| Compound | Cell line | $IC_{50}$ (μM) |
|---|---|---|
| SKI 178 | MCF10A | 1.83 |
| | MCF10A *CDH1*$^{-/-}$ | 1.75 |
| MP A08 | MCF10A | ≥50 |
| | MCF10A *CDH1*$^{-/-}$ | ≥50 |
| SLC5111312 | MCF10A | 7.04 |
| | MCF10A *CDH1*$^{-/-}$ | 4.99 |
| L-cycloserine | MCF10A | ≥50 |
| | MCF10A *CDH1*$^{-/-}$ | ≥50 |
| GW4869 | MCF10A | ≥50 |
| | MCF10A *CDH1*$^{-/-}$ | 6.13 |
| W123 | MCF10A | ≥50 |
| | MCF10A *CDH1*$^{-/-}$ | 43.40 |
| JTE-013 | MCF10A | 46.55 |
| | MCF10A *CDH1*$^{-/-}$ | 44.54 |
| VPC 23019 | MCF10A | ≥50 |
| | MCF10A *CDH1*$^{-/-}$ | ≥50 |
| Fingolimod | MCF10A | 3.35 |
| | MCF10A *CDH1*$^{-/-}$ | 2.75 |

**Table S6.** Cell viability ratios following inhibition of endocytosis or Src family kinases.

| Compound | Concentration (μM) | Ratio of MCF10A *CDH1*$^{-/-}$ to MCF10A viability |
|---|---|---|
| PACOCF3 | 0.2 | 1.01 |
| | 0.39 | 0.97 |
| | 0.78 | 1.00 |
| | 1.56 | 1.01 |
| | 3.13 | 0.98 |
| | 6.25 | 1.03 |
| | 12.5 | 1.00 |
| | 25 | 0.97 |
| | 50 | 1.14 |
| Dyngo-4a | 0.64 | 1.02 |
| | 0.98 | 1.04 |
| | 1.51 | 1.01 |
| | 2.32 | 1.06 |
| | 3.57 | 0.99 |
| | 5.49 | 1.07 |
| | 8.45 | 1.07 |
| | 13 | 1.05 |
| | 20 | 1.07 |
| EGA | 0.2 | 1.00 |
| | 0.39 | 0.98 |
| | 0.78 | 0.97 |
| | 1.56 | 0.99 |
| | 3.13 | 0.97 |
| | 6.25 | 0.93 |
| | 12.5 | 0.86 |
| | 25 | 0.83 |
| | 50 | 0.78 |
| Lck inhibitor | 0.05 | 1.03 |
| | 0.11 | 1.01 |
| | 0.21 | 1.01 |
| | 0.42 | 0.99 |
| | 0.84 | 0.92 |
| | 1.69 | 1.00 |
| | 3.38 | 0.94 |
| | 6.75 | 0.99 |
| | 13.5 | 0.90 |
| Bosutinib | 0.2 | 0.98 |
| | 0.39 | 1.02 |
| | 0.78 | 0.87 |
| | 1.56 | 0.85 |
| | 3.13 | 0.90 |
| | 6.25 | 0.87 |
| | 12.5 | 0.91 |
| | 25 | 0.79 |
| | 50 | 0.81 |

**Table S7.** $IC_{50}$ values from inhibition of endocytosis or Src family kinases.

| Compound | Cell line | $IC_{50}$ (μM) |
|---|---|---|
| PACOCF3 | MCF10A | ≥50 |
| | MCF10A *CDH1*$^{-/-}$ | ≥50 |
| Dyngo-4a | MCF10A | ≥50 |
| | MCF10A *CDH1*$^{-/-}$ | ≥50 |
| EGA | MCF10A | ≥50 |
| | MCF10A *CDH1*$^{-/-}$ | ≥50 |
| Lck inhibitor | MCF10A | ≥50 |
| | MCF10A *CDH1*$^{-/-}$ | ≥50 |
| Bosutinib | MCF10A | 3.16 |
| | MCF10A *CDH1*$^{-/-}$ | 2.55 |

**Table S8.** Targets of Src family kinase inhibitors. Targets are ordered in ascending order of $IC_{50}$ values.

| Compound | Target | $IC_{50}$ (nM) |
|---|---|---|
| PP1 | Lck | 5 |
| | Fyn | 6 |
| | Hck | 20 |
| | c-Src | 170 |
| PP2 | Lck | 4 |
| | Fyn | 5 |
| | Hck | 5 |
| | c-Src | 33 |
| SU6656 | Yes | 20 |
| | Lyn | 130 |
| | Fyn | 170 |
| | c-Src | 280 |
| Bosutinib | c-Src | 1.2 |
| Lck inhibitor | Lck | 1 |
| | c-Src | 70 |

**Table S9.** Cell viability ratios following inhibition of autophagy.

| Compound | Concentration (μM) | Ratio of MCF10A *CDH1*$^{-/-}$ to MCF10A viability |
|---|---|---|
| STF-62247 | 0.2 | 0.93 |
| | 0.39 | 1.08 |
| | 0.78 | 0.93 |
| | 1.56 | 0.86 |
| | 3.13 | 0.89 |
| | 6.25 | 0.89 |
| | 12.5 | 0.72 |
| | 25 | 0.63 |
| | 50 | 0.64 |

**Table S10.** $IC_{50}$ values from inhibition of autophagy.

| **Compound** | **Cell line** | **$IC_{50}$ (μM)** |
|---|---|---|
| STF-62247 | MCF10A | ≥50 |
| | MCF10A *CDH1*$^{-/-}$ | 23.82 |

**Table S11.** MCF10A cell viability ratios following treatment with drug combinations.

| Drug combination | Concentration (μM) | Ratio of MCF10A *CDH1*$^{-/-}$ to MCF10A viability |
|---|---|---|
| PF-543/chloroquine | 0.2/0.2 | 0.89 |
| | 0.39/0.39 | 0.89 |
| | 0.78/0.78 | 0.83 |
| | 1.56/1.56 | 0.81 |
| | 3.13/3.13 | 0.67 |
| | 6.25/6.25 | 0.64 |
| | 12.5/12.5 | 0.75 |
| | 25/25 | 1.19 |
| PF-543/chlorpromazine | 0.2/0.1 | 0.97 |
| | 0.39/0.2 | 0.91 |
| | 0.78/0.39 | 0.93 |
| | 1.56/0.78 | 0.86 |
| | 3.13/1.56 | 0.73 |
| | 6.25/3.13 | 0.59 |
| | 12.5/6.25 | 0.64 |
| | 25/12.5 | 0.78 |
| PF-543/atorvastatin | 0.2/0.31 | 0.90 |
| | 0.39/0.63 | 0.83 |
| | 0.78/1.25 | 0.85 |
| | 1.56/2.5 | 0.82 |
| | 3.13/5 | 0.75 |
| | 6.25/10 | 0.76 |
| | 12.5/20 | 0.93 |
| | 25/40 | 0.87 |

**Table S12.** NCI-N87 **c**ell viability ratios following treatment with candidate synthetic lethal compounds.

| Compound | Concentration (μM) | Ratio of NCI-N87 *CDH1*$^{-/-}$ to NCI-N87 viability |
|---|---|---|
| PF-543 | 0.2 | 1.00 |
| | 0.39 | 1.02 |
| | 0.78 | 1.05 |
| | 1.56 | 0.98 |
| | 3.13 | 1.04 |
| | 6.25 | 1.00 |
| | 12.5 | 0.93 |
| | 25 | 1.16 |
| | 50 | 1.17 |
| PP1 | 0.16 | 1.12 |
| | 0.31 | 1.21 |
| | 0.63 | 1.23 |
| | 1.25 | 1.30 |
| | 2.5 | 1.40 |
| | 5 | 1.16 |
| | 10 | 0.92 |
| | 20 | 0.91 |
| Chlorpromazine | 0.39 | 1.01 |
| | 0.78 | 1.04 |
| | 1.56 | 0.96 |
| | 3.13 | 0.94 |
| | 6.25 | 0.93 |
| | 12.5 | 0.86 |
| | 25 | 0.77 |
| | 50 | 0.90 |
| Chloroquine | 0.39 | 0.98 |
| | 0.78 | 1.01 |
| | 1.56 | 0.99 |
| | 3.13 | 1.00 |
| | 6.25 | 0.96 |
| | 13 | 0.92 |
| | 25 | 0.92 |
| | 50 | 0.94 |

**Table S13.** $IC_{50}$ values for candidate synthetic lethal compounds in NCI-N87 isogenic cells.

| Compound | Organoid line | $IC_{50}$ (μM) |
|---|---|---|
| PF-543 | NCI-N87 | 33.71 |
| | NCI-N87 *CDH1*$^{-/-}$ | 35.88 |
| PP1 | NCI-N87 | 1.50 |
| | NCI-N87 *CDH1*$^{-/-}$ | 3.52 |
| Chlorpromazine | NCI-N87 | 42.63 |
| | NCI-N87 *CDH1*$^{-/-}$ | 35.39 |
| Chloroquine | NCI-N87 | ≥50 |
| | NCI-N87 *CDH1*$^{-/-}$ | ≥50 |

**Table S14.** Gastric organoid viability ratios following treatment with candidate synthetic lethal compounds.

| Compound | Concentration (μM) | Ratio of *Cdh1*$^{-/-}$ organoid to *Cdh1*$^{+/+}$ organoid viability |
|---|---|---|
| PF-543 | 0.39 | 0.48 |
| | 0.78 | 0.52 |
| | 1.57 | 0.38 |
| PP1 | 6.5 | 0.53 |
| | 13 | 0.54 |
| | 26 | 0.44 |
| Chlorpromazine | 6.25 | 0.35 |
| | 12.5 | 0.53 |
| | 25 | 0.70 |
| Chloroquine | 12.5 | 0.40 |
| | 25 | 0.39 |
| | 50 | 0.49 |

**Table S15.** $IC_{50}$ values for candidate synthetic lethal compounds in *Cdh1*$^{+/+}$ and *Cdh1*$^{fl/fl}$ gastric organoids.

| **Compound** | **Organoid line** | **$IC_{50}$ (μM)** |
|---|---|---|
| PF-543 | *Cdh1*$^{+/+}$ organoids | 1.84 |
| | *Cdh1*$^{fl/fl}$ organoids | 0.38 |
| PP1 | *Cdh1*$^{+/+}$ organoids | 27.42 |
| | *Cdh1*$^{fl/fl}$ organoids | 9.75 |
| Chlorpromazine | *Cdh1*$^{+/+}$ organoids | 7.24 |
| | *Cdh1*$^{fl/fl}$ organoids | 0.02 |
| Chloroquine | *Cdh1*$^{+/+}$ organoids | 31.76 |
| | *Cdh1*$^{fl/fl}$ organoids | 0.84 |

**Table S16.** Mammary organoid viability ratios following treatment with candidate synthetic lethal compounds.

| Compound | Concentration (μM) | Ratio of *Cdh1*$^{fl/fl}$ organoid to *Cdh1*$^{fl/fl}$ organoid viability |
|---|---|---|
| PF-543 | 0.39 | 0.62 |
| | 0.78 | 0.74 |
| | 1.57 | 0.52 |
| PP1 | 6.5 | 1.01 |
| | 13 | 0.99 |
| | 26 | 0.77 |
| Chlorpromazine | 6.25 | 1.09 |
| | 12.5 | 0.70 |
| | 25 | 0.56 |
| Chloroquine | 12.5 | 1.22 |
| | 25 | 1.09 |
| | 50 | 0.57 |

**Table S17.** $IC_{50}$ values for candidate synthetic lethal compounds in *Cdh1*$^{+/+}$ and *Cdh1*$^{fl/fl}$ mammary organoids.

| **Compound** | **Organoid line** | **$IC_{50}$ (μM)** |
|---|---|---|
| PF-543 | *Cdh1*$^{+/+}$ organoids | 46.76 |
| | *Cdh1*$^{fl/fl}$ organoids | 19.94 |
| PP1 | *Cdh1*$^{+/+}$ organoids | 42.51 |
| | *Cdh1*$^{fl/fl}$ organoids | 24.86 |
| Chlorpromazine | *Cdh1*$^{+/+}$ organoids | 133.13 |
| | *Cdh1*$^{fl/fl}$ organoids | 66.10 |
| Chloroquine | *Cdh1*$^{+/+}$ organoids | 167.49 |
| | *Cdh1*$^{fl/fl}$ organoids | 87.03 |
